# Macrophages bend long fibres with flexural rigidity lower than 3 mN·nm^2^ to avoid frustrated phagocytosis

**DOI:** 10.1186/s12989-026-00666-9

**Published:** 2026-03-18

**Authors:** Dirk Broßell, Asmus Meyer-Plath, Oliver Gräb, Elisabeth Heunisch, Kerstin Kämpf, Andrea Haase, Martin Wiemann

**Affiliations:** 1 Divison 4: Hazardous Substances and Biological Agents, Federal Institute for Occupational Safety and Health (BAuA), Nöldnerstr. 40-42, 10317 Berlin, Germany; 2 Institut für Medizinische Physik und Biophysik, Charité – Universitätsmedizin Berlin, Corporate Member of Freie Universität Berlin and Humboldt- Universität zu Berlin, Charitéplatz 1, 10117 Berlin, Germany; 3 IBE R&D Institute for Lung Health gGmbH, Mendelstr. 11, 48149 Münster, Germany; 4 Department of Chemical and Product Safety, German Federal Institute for Risk Assessment (BfR), Max-Dohrn-Straße 8-10, 10589 Berlin, Germany; 5 Institute of Pharmacy, Freie Universität Berlin, Königin-Luise-Straße 2-4, 14195 Berlin, Germany; 6 Department of Chemical Sciences, University of the Western Cape (UWC), 7535 Bellville, South Africa

**Keywords:** Fibre phagocytosis, Flexural rigidity, Fibre-pathogenicity paradigm, Critical rigidity threshold, Time-lapse microscopy

## Abstract

**Background:**

It is an established toxicological principle that the inhalation pathogenicity of respirable and biodurable fibres is caused by excessive fibre length as alveolar macrophages fail to uptake and remove such fibres. However, studies on carbon nanotubes showed that this principle needs revision, as thin, flexible variants showed reduced fibre-specific toxicity. One potential explanation is that the low flexural rigidity of thin fibres enables macrophages to bend and internalize even those that are long relative to the cell size. To evaluate this proposed “rigidity hypothesis,” the mechanisms governing the uptake of flexible long fibres that determine a critical threshold value for flexural rigidity require clarification.

**Methods:**

We exposed NR8383 rat alveolar macrophages to three silver nanowire variants differing in diameter and length. Time-lapse microscopy captured fibre uptake processes. Successful internalization of long fibres was found to require fibre bending during uptake. A mechanical model was developed by combining established cytoskeletal biophysics with the observed fibre deformation dynamics. As flexural rigidity describes fibre behaviour under load, our model estimated rigidity by reproducing the observed bent fibre shape. By defining limit cases for physically ‘weak’ and ‘strong’ NR8383 macrophages, i.e., assuming upper bounds on the forces generated by their cytoskeletal nanomachinery, our model enabled us to derive a range for the critical fibre rigidity threshold.

**Results and conclusion:**

A macrophage was observed bending an exceptionally long fibre (~ 140 μm) first into an arc and then a spiral for full internalization, initiated by a pseudopod extending along the fibre and buckling the internalized segment. Our model can reproduce such behaviour. It yielded a flexural rigidity of 20 mN·nm² for this fibre. Predicted critical rigidity limits for fibres that just fit into NR8383 macrophages range from 3 to 62 mN·nm². Using the conservative lower bound, long and biodurable fibres with a rigidity lower than 3 mN·nm² are expected to be readily phagocytized by this cell line. Although this rigidity scale may not be directly translatable to human alveolar macrophages, our experimental findings and their modeling emphasize the key role of rigidity in fibre–cell interactions. Fibre rigidity is therefore central for material safety aspects and sustainable product design.

**Supplementary Information:**

The online version contains supplementary material available at 10.1186/s12989-026-00666-9.

## Background

Respired biopersistent fibres such as asbestos were found to cause severe pulmonary diseases and led to proposing the fibre-pathogenicity paradigm [1–3]. Alveolar macrophages attempt to ingest and remove respired micro-organisms and dust particles [4, 5]. This lung clearance mechanism, which is effective on granular (low-aspect ratio) particles, can fail for fibres [6]. Fibres persisting in lung tissue can trigger chronic inflammatory processes leading to lung fibrosis, lung cancer and to mesothelioma [7, 8]. Phagocytized objects are enclosed in a phagolysosome and degraded via oxidative and/or enzymatic processes, or, if non-degradable, transported from the lung parenchyma via the mucociliary escalator to the larynx for removal from the body [9, 10]. Macrophages tend to stall the uptake of fibres longer than their own size if the cell membrane cannot sufficiently be deformed to enclose the whole fibre. This so-called ‘frustrated phagocytosis’ leaves the cell in a penetrated state [11, 12]. For occupational exposure to biodurable fibres longer than 5 μm and thinner than 3 μm—toxicologically *‘critical fibres’*—many countries have established strict legal regulations, as such fibres are considered harmful due to their respirability, biodurability, and length [13].

**Fig. 1:**
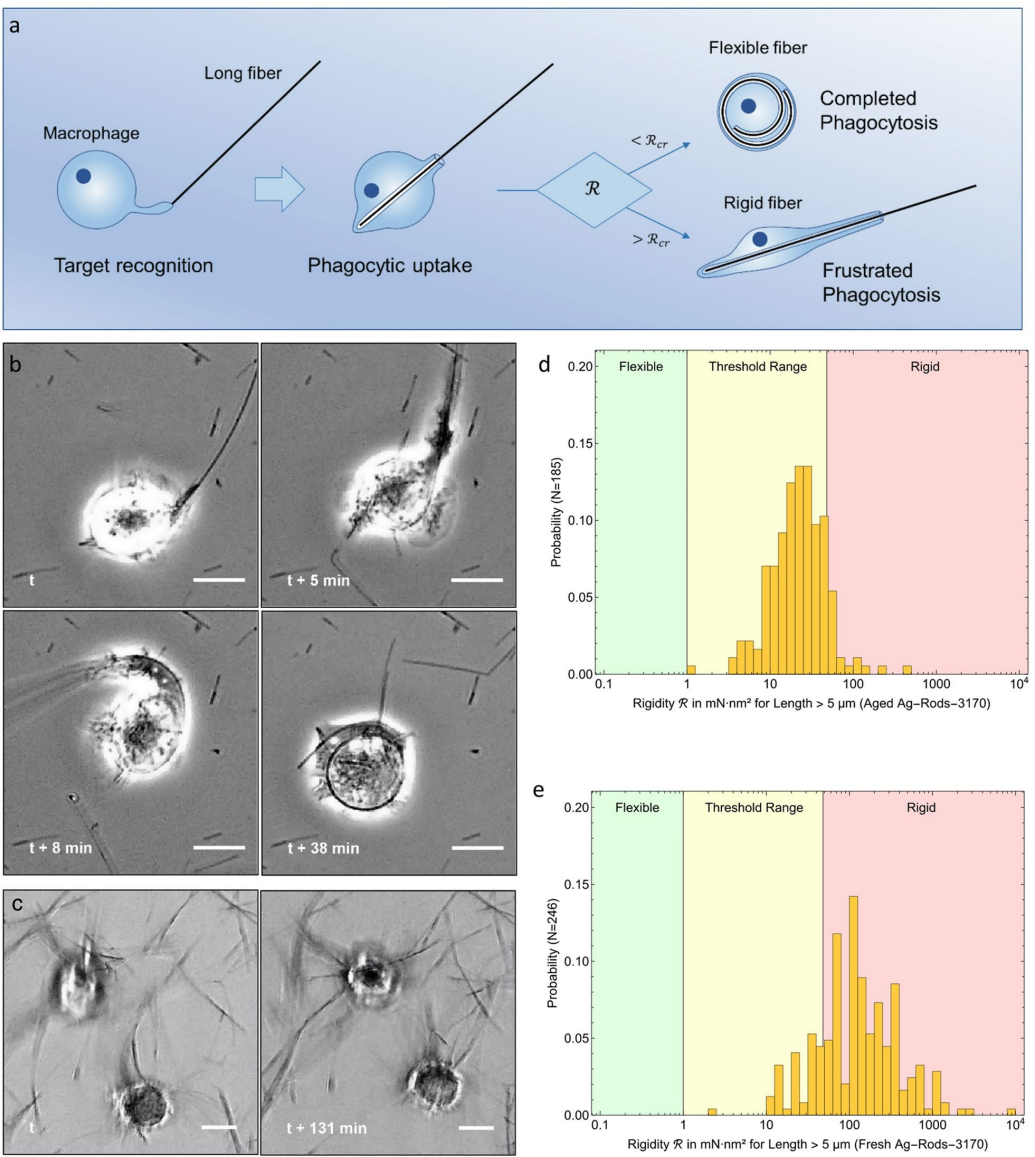
Outline of the ‘rigidity hypothesis’ and supporting observations linking fibre rigidity to distinctly different behaviours during phagocytosis of long fibres. **a**, Visualization of events observed during phagocytosis of long fibres leading to different outcomes: a macrophage contacts the fibre tip with a pseudopodium, then takes it up into a membrane pouch. If the fibre is flexible ($\:\mathcal{R}$ < $\:{\mathcal{R}}_{cr})\:$the macrophage can complete phagocytosis. Frustrated phagocytosis occurs when fibre rigidity $\:\mathcal{R}$ exceeds the critical rigidity threshold value $\:{\mathcal{R}}_{cr}\:$($\:\mathcal{R}$ > $\:{\mathcal{R}}_{cr})\:$and is too long to be taken up completely. **b**, Time-lapse capture of the completion of phagocytosis of a very long fibre of the aged Ag-Rods-3170 variant by a NR8383 macrophage. The fibre is first engaged and partly taken up at time *t* followed by a pseudopod extending along the fibre shaft (*t* + 5 min) that subsequently arcs and bends the fibre (*t* + 8 min), leading to full internalization (*t* + 38 min). This observation corroborates the stated rigidity hypothesis. Scale bars represent 10 µm. **c**, Time-lapse captures taken over more two hours apart (*t*, *t* + 131 min) of two macrophages unable to take up more rigid fresh Ag-Rods-3170. **d**, Rigidity histogram calculated for 185 critical (length > 5 µm) aged Ag-Rod-3170 nanowires, indicating that a large portion ( ~ 88%) of critical nanowires exhibited rigidity within the threshold range defined by [25]., **e**, For fresh Ag-Rods-3170 nanowires, only a small portion exhibited rigidity within the threshold range and a large portion ( ~ 82%) was rigid

Observations of strong correlations between fibre properties and adverse health effects have been reported for a wide variety of fibre materials [14–16], However, a specific class of nanoscale materials, carbon nanotubes (CNTs), was found not to be consistently toxic despite the fibre-pathogenicity paradigm’s prediction [17, 18]. One reason for reduced CNT toxicity was attributed to the fact that many CNT materials were toxicologically tested in agglomerate state as CNTs can already be synthesized as highly entangled agglomerates, can spontaneously re-agglomerate after dispersion, or, if thin and flexible, can self-tangle into granules. Such granular agglomerates are not expected to show fibre-specific toxicity. For individual thin fibres of low flexural rigidity, a term shortened to ‘rigidity’ subsequently, it was suggested that macrophages may compactify such fibres during internalization [19]. This notion was later supported by observations of initially straight silver nanowires appearing curled inside J774A.1 macrophages [20]. This so-called ‘rigidity hypothesis’ was proposed as an extension to the fibre-pathogenicity paradigm [20–24]. It states that the excessive length and rigidity of a chemically inert fibre has the potential to induce carcinogenicity, provided that the fibre is respirable and biodurable. The rigidity hypothesis posits that phagocytosis of long fibres that significantly exceed the dimensions of macrophages can fail if their flexural rigidity $\:\mathcal{R}$ exceeds a critical threshold $\:{\mathcal{R}}_{cr}$ (see Fig. 1a). By contrast, fibres with a rigidity below this threshold can be completely internalised. However, the mechanisms governing the uptake of long fibres and the definition of the critical rigidity threshold are currently only understood incompletely.

For clarity, flexural rigidity is defined as the product of the bending modulus and the second moment of area of the fibre cross-section. For compact cylindrical fibres, the latter scales with the fourth power of the fibre diameter. This means that thinner fibres exhibit significantly greater flexibility and may be deformed by the forces generated by macrophages during internalisation.

Against this mechanistic background, the rigidity hypothesis provides a framework for fibre toxicology in which toxicological differentiation is based on measurable physical properties rather than on fibre material identity. Specifically, the distributions of four key parameters – length, diameter, bending modulus and biodurability – can be utilised to compare fibre ensembles with in vivo tested reference materials, such as asbestos or carbon nanotubes.

By introducing flexural rigidity, geometric fibre properties (length and diameter) can be considered separately from material-specific properties (biodurability and bending modulus), thereby extending the classical fibre-pathogenicity paradigm. Despite this strong diameter dependence, material-specific compositional and structural characteristics remain relevant for determining which fibres exceed the critical rigidity threshold. Establishing this hypothesis will therefore require an improved understanding of fibre uptake mechanisms and macrophage-specific bending force limits.

This study aims at investigating the role of rigidity in the failure of fibre phagocytosis using an experimental in vitro system of NR8383 macrophages exposed to three silver nanowires with different length and width distributions. Each batch exhibited a distribution of different rigidities and one contained a large fraction of fibres supposedly flexible enough for phagocytosis. Time-lapse videos of macrophages interacting with administered fibres enabled to study both the dynamics of successful and failing phagocytosis.

We were able to record how a macrophage bent a fibre much longer than its own size into an arc and subsequently internalized it. Drawing on literature knowledge about cytoskeletal nanomachinery, a mathematical model is proposed providing insight into fibre bending and the rigidity thresholds associated with frustrated phagocytosis.

## Materials and methods

In this study, NR8383 rat alveolar macrophages were exposed under submerged conditions to three silver nanowire variants: fresh Ag-NWs-40, fresh Ag-Rods-3170, and aged Ag-Rods-3170. The nanowires differed in length and rigidity, as shown later. Interactions and phagocytosis processes of macrophages with these nanowire materials were captured in a series of time-lapse videos with a rate of one frame per minute. This way, the dynamics of the uptake of flexible and rigid silver fibres were recorded in detail.

### Fibre materials

The Ag-NWs-40 material was supplied by ACS Materials, California, USA (type Agnw-40) with polyvinylpyrrolidone functionalization and dispersed in water. The manufacturer specified a diameter of $\:40\:\mathrm{n}\mathrm{m}$, a length of $\:20-60\:{\upmu}\mathrm{m}$ and a purity of 99.5%, and described the fibre cross-section as pentagonal, which may indicate a crystalline material. The Ag-Rods-3170 material was supplied by NANOGAP Europe, Spain, with polyvinylpyrrolidone functionalization and dispersed in water (type NGAP NF Ag-3170-W). The manufacturer specified a diameter in the range of 60–80 nm and a length of 30 μm. The material is described to be synthesized using crystalline silver clusters that catalyse the growth of crystalline fibres in solution [Patent WO 2011/113914]. After initially using a fresh batch of Ag-Rods-3170, the material was again used to generate a second batch after 127 days in storage.

### Fibre material characterization

For scanning electron microscopical visualization of the silver nanowires and characterization on the micrographs, the original aqueous fibre suspensions were mixed with isopropanol (99.95%). For the Ag-Rods-3170 variants, $\:4\:{\upmu}\mathrm{L}$ was dispersed in $\:2\:\mathrm{m}\mathrm{L}$ isopropanol. $\:10\:{\upmu}\mathrm{l}$ of Ag-NWs-40 suspension was mixed with 4 mL isopropanol. Mixing steps were conducted in N_2_-atmosphere. Subsequently, a 20 µL-droplet was pipetted onto a clean Si-Wafer. The wafer was then dried. SEM analysis was conducted using a Hitachi SU8230 scanning electron microscope (SEM). Micrographs were acquired at random locations on the sample. For the fibre characterization, the particle analysis software “FibreDetect” was used that allows the tracking of the fibre longitudinal axis and the adjustment of the diameter along the fibre shaft [26]. The spline pixel length and diameter pixel length were then converted to nanometres using the pixel size known from the image acquisition.

At least 300 fibres of each sample were measured with respect to fibre length and diameter. The whole procedure is further described in the Test No. 125 “Particle Size and Size Distribution of Nanomaterials” of the OECD Guidelines for the Testing of Chemicals [27].

The fibre width distributions of the characterized ensembles were used to derive the rigidity distributions. Rigidity $\:\mathcal{R}$ depends on bending modulus *E*_*b*_ and axial fibre cross section *I*_*a*_, which, for cylindrical fibres, is determined by the outer diameter *D*:

1 $${\mathcal{R}} = I_{a} \cdot E_{b} = \frac{\pi }{{64}}D^{4} \cdot E_{b} $$

### Cell culture

Rat alveolar macrophages (NR8383) were obtained from the American Type Culture Collection (ATCC) and cultured in accordance with the methods described by Wiemann et al. [28]. These cells are highly motile and can ingest foreign objects, including nanomaterials and nanofibres, even under serum-free conditions. This property makes them a suitable in vitro model for studying particle–macrophage interactions in the alveolar environment, enabling the comparison of materials with different surface functionalisation. The biological responses of NR8383 cells to nanomaterials are well documented and have been shown to correlate with in vivo outcomes [28]. This supports their use as a predictive model and contributes to the reduction and refinement of animal experiments. To date, no comparably validated in vitro model based on human alveolar macrophages is available.

The cells were cultured in an F-12 K medium supplemented with 2 mM glutamine, penicillin (100 U/mL) and streptomycin (10 µg/mL) (all from PAN Biotech GmbH, Germany), as well as 15% (v/v) fetal calf serum. The cultures were grown in 175 cm² flasks at 37 °C in a humidified atmosphere containing 5% CO₂, and fed by replacing half of the medium twice per week.

### Exposure to nanofibres and time-lapse video imaging

A total of 32,000 NR8383 cells in 300 µL of serum-free F-12 K medium were seeded into each compartment of a fibronectin-coated 35 mm µ-dish (Quad, ibe-treat, No. 80416; ibidi Gräfelfing, Germany). Cells were allowed to attach for 24 h under cell culture conditions. Then, 300 µL medium containing 4 µg of either silver-nanowire quality per mL F-12 K medium were added, to obtain a final concentration of 2 µg/mL. The µ-dish was transferred into the observation chamber of a Nikon BioStation (pre-warmed to 37 °C, and continuously perfused with warm, humidified air containing 5% CO_2_). The µ-dish was allowed to equilibrate for ca. 1 h to achieve optically stable conditions. Regions of interest were focussed using a 20x objective and phase contrast settings. Time-lapse recording was started with a frame rate of 1/min and continued for indicated times.

## Results and discussion

### Bending of long silver nanowires during phagocytosis

We observed a macrophage manipulating, bending, and ultimately internalizing a fibre exceeding its own size (Fig. 1b, Supplemental Video 1). This silver nanowire from the aged Ag-Rods-3170 exhibited a length of 139 ± 3 μm, as measured before and after its uptake into the cell. The macrophage recognised the long fibre that settled onto the bottom of the cell culture dish, took hold of the fibre tip using an extending pseudopod and started to pull the fibre in. This initial uptake stage stalled when the first part of the fibre spanned over the whole interior cell and its tip poked and deformed the cell membrane (Fig. 1b, t). At this stage, the internalized portion of the fibre measured ca. 26 μm, which is roughly twice the size of a typical NR8383 macrophage in its detached and rounded state (12.5 –12.8 μm) [28]. The cell then continued to extend a ca. 25 μm long pseudopod along the fibre shaft (Fig. 1b, t + 5 min). Note that the cell could accommodate more than 50 μm of the fibre shaft with cell body and pseudopod. Remarkably enough, the pseudopod subsequently arced and bent the fibre (Fig. 1b, t + 8 min). As a seemingly stiff cell membrane forced the fibre to follow the membrane’s curvature during internalization, the progressively ingested fibre was wound up into spiral spring shape while the macrophage had assumed a diameter of ca. 20 ± 1 μm (Fig. 1b, t + 38 min). The duration from target recognition, the end of the initial uptake stage and pseudopod extension was approximately 10 min. Fibre bending and subsequent full internalization lasted ca. 46 min.

In contrast, interactions of macrophages with the fresh variant of Ag-Rods-3170 nanowires were characterized by a continuous struggle to engage and take up fibres in the cells’ immediate surroundings (Fig. 1c, Supplementary Video 2). The macrophages constantly grabbed and moved nanowires but did not internalize them completely.

**Table 1:** Material and geometric properties for used critical fibres (L > 5 μm)

Material		Material properties		Properties of long and rigid fibres	
	Sample size	Fraction of critical fibres	Geometric mean length	Geometric mean width	Geometric mean rigidity*
	No. of fibres	%	[µm]	[nm]	[mN nm2]
Ag-NWs-40 *NANOGAP Europe*	371	50	10 ± 4	53 ± 7	$\:{30}_{-16}^{+28}$
Ag-Rods-3170 *ACS Materials*	339	72	12 ± 11	78 ± 18	$\:{148}_{-115}^{+294}$
Ag-Rods-3170 (aged for 127 days)	341	54	10 ± 4	49 ± 7	$\:{23}_{-14}^{+15}$

*assuming a bending modulus of 80 ± 20 GPa [29]

According to the rigidity hypothesis, the evident difference in macrophage behaviour can be explained by differences in rigidity between the two silver nanowire variants, as determined by material and fibre characterization using scanning electron microscopy (Table 1, Supplemental information Figure S1). Fresh Ag-Rods-3170 nanowires contained a critical fibre (length > 5 μm) fraction of 72%, with widths of 78 ± 24 nm and lengths of 15 ± 9 μm, occasionally exceeding 100 μm. Length values likely represent underestimations, since some fibres appeared to break during sample preparation, while widths remained unaffected. Rigidity was calculated (Eq. 1) assuming a bending modulus of 80 ± 20 GPa [29]. Critical fibres of fresh Ag-Rods-3170 exhibited a rigidity of 148$\:{\:}_{-115}^{+294}$ mN·nm². After 127 days of storage, the aged Ag-Rods-3170 nanowires had reduced widths (49 ± 10 nm) and lengths (10 ± 3 μm), with a critical fibre fraction of ~ 54% and 23$\:{\:}_{-14}^{+26}$ mN·nm². Very long fibres (> 50 μm) as observed with phase contrast microscopy were absent, either because they were extremely rare or broke during sample preparation.

Intraperitoneal injection of suspensions of different MWCNT materials in rodents that were kept for 24 months showed fibre-specific toxicity for critical fibres exhibiting different geometric mean diameters except thinnest with a diameter of 37$\:{\:}_{-11}^{+17}\:\mathrm{n}\mathrm{m}$ [25], which would yield a rigidity of 7$\:{\:}_{-6}^{+31}$ mN·nm², assuming a bending modulus of E_b_=78±15 GPa [22]. Of note, the specified geometric standard deviation was used to calculate the limits of 68.3% confidence intervals given as asymmetric errors here. The referenced study remains the only one known to the authors that reported results on single fibres from in vivo studies in a presumed critical rigidity threshold range. The rigidity distribution of the aged silver nanowires (Fig. 1d) largely overlapped with this estimated rigidity threshold range, whereas the fresh variant (Fig. 1e) extended well into the rigid range. Consequently, phagocytosis resistance was more likely for fresh than aged Ag-Rods-3170, explaining the observed difference in macrophage behaviour.

**Fig. 2:**
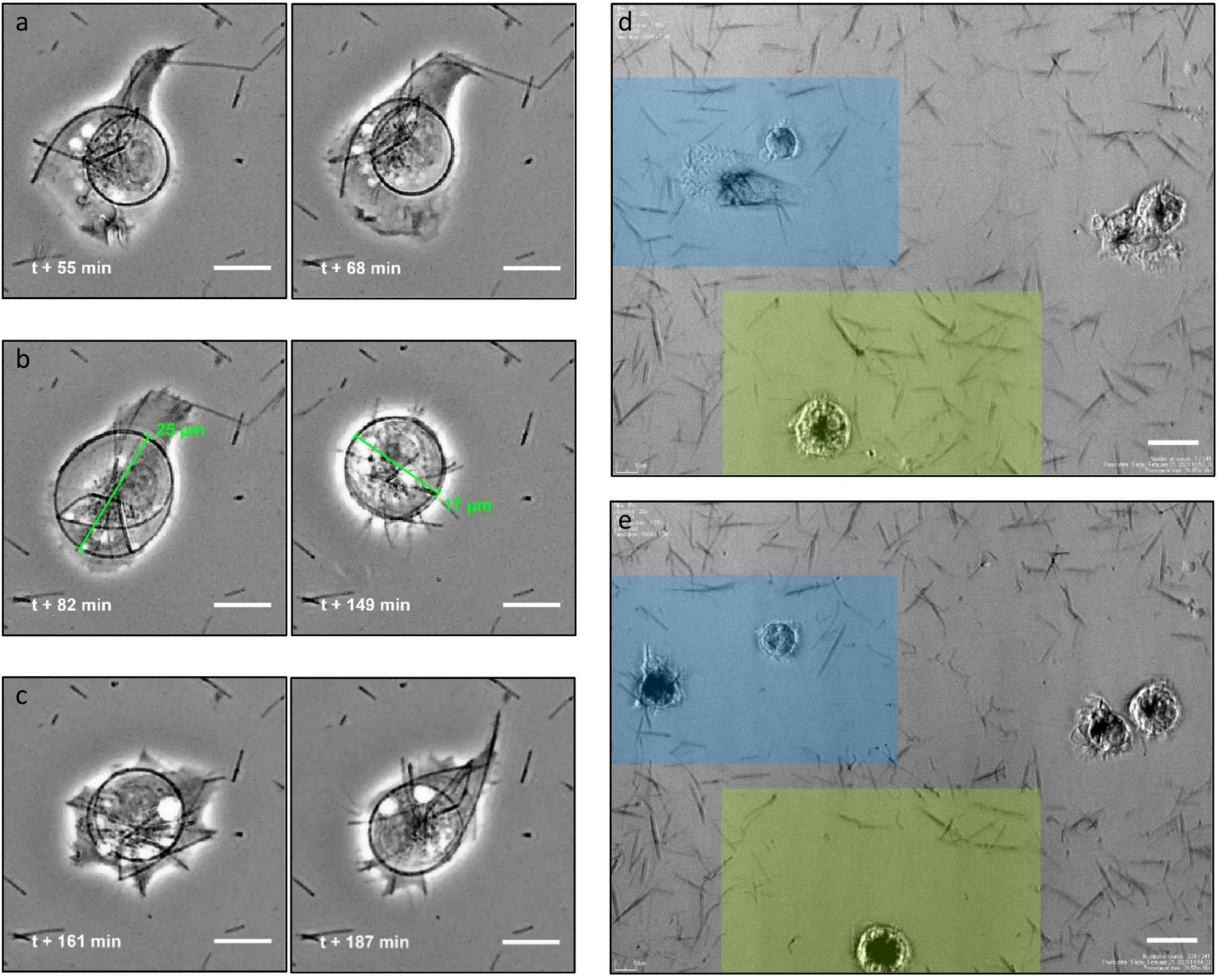
Observations of macrophage-fibre interactions. **a**, After taking up the long silver nanowire of the aged AG-Rods-3170 variant, the macrophage continued to take up a fibre with an extended pseudopod (left panel), initiating bending which results in higher curvature of the fibre (right panel). **b**, Afterwards, the cell increased its size to ca. 25 μm and the fibre spring extended (left panel). The macrophage attempted to constrain the fibre by decreasing its size to ca. 17 μm (right panel). **c**, The cell maintained the shape of the fibre spiral while still actively extending pseudopodia (left panel), but after more than three hours after the initial bending event at time $\:t$, the macrophage seemed to allow unwinding of the nanowire with the tips poking and stretching the cell membrane (right panel). Scale bars in a-c represent 10 μm. **d** and **e**, Early time-point (top panel) and late time-point (bottom panel) of macrophages cleansing the fibre-covered surface from nanowires from the Ag-NWs-40 variant, taken ca. 4 h apart. The highlighted areas, covered with nanowires at the beginning, were cleared of fibres during the observation time. The macrophages visibly appear to have increased their fibre load. Scale bars 20 μm

Containing the spiral fibre was a continuous struggle for the macrophage, as illustrated in Fig. 2a-c. Throughout the observation time after the bending event, the fibre retained its elastic properties. Meanwhile, the same macrophage continued ingesting additional fibres for about 20 min, including bending a much shorter one (Fig. 2a). Whenever cortical strain of the cell membrane relaxed, the spiral fibre expanded (Fig. 2b, t + 82 min), prompting the macrophage to counteract by forcing it back into its previous shape (Fig. 2b, t + 149 min). The cell continuously extended several pseudopodia (Fig. 2c, t + 161 min), thus retaining its functionality. Ultimately, however, it failed to maintain control, and the spiral expanded with both tips protruding into the cell membrane (Fig. 2c, t + 187 min).

Aged Ag-Rods-3170 displayed similar properties to the Ag-NWs-40 variant, which had widths of 52 ± 9 nm, lengths of 11 ± 3 μm, and a rigidity of 30$\:{\:}_{-16}^{+28}$ mN·nm². Very long fibres, as observed in fresh Ag-Rods-3170, were absent. As illustrated in Fig. 2d, macrophages migrated across the fibre-loaded surfaces over the course of several hours (~ 3:50 h), removing numerous fibres in the process (Supplementary Video 3). Throughout these observations, no bending events occurred, indicating that the fibres could be internalized without macrophages inducing morphological changes.

### Actomyosin network orchestrates fibre bending

The observation of arcing pseudopods that actively bend fibres motivated us to theoretically consider the origin of dynamic deformation of the cytoskeleton. Based on published data, we are proposing how the cytoskeletal nanomachinery drives pseudopod deformations in order to estimate the resulting forces and their effect on fibres.

**Fig. 3:**
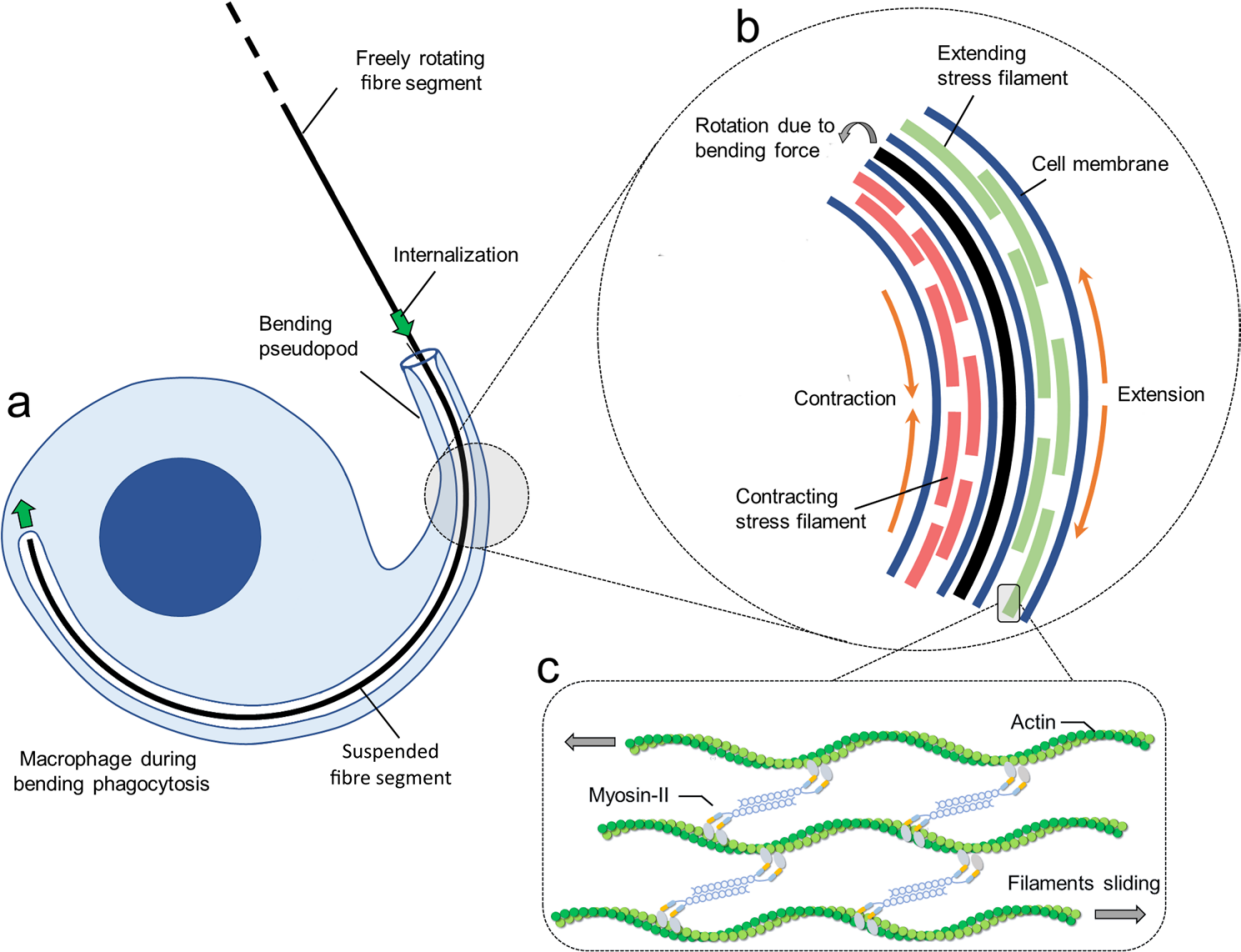
Molecular nanomachinery driving pseudopod arcing and fibre bending. **a**, A fibre is bend into an arc by a pseudopod extended by the macrophage that suspends the fibre. **b**, Within the pseudopod, stress filaments work together in causing an elastic deformation that drives rotational movement of its tip and bending of the suspended fibre. **c**, Actomyosin structures in the stress filaments generate its contraction/extension [30–34]

A biophysical mechanism underlying pseudopod arcing and fibre bending is outlined in a top-down approach (Fig. 3), first figuring the macrophage in the process of bending (Fig. 1b, t + 8 min): a partially internalized fibre is bent via its arcing pseudopod (Fig. 3a).

Bending occurs along the fibre segment that is enclosed within the tubular pseudopod but still outside the main cell body. Internalization of elongated pathogens has been reported to be facilitated by actin clearance at the pseudopod base, which allows membrane invagination into the cytoplasm [30]. While the precise mechanism driving continuous inward transport of a fibre remains uncertain, evidence from particle uptake studies suggests that forces are generated by actin–myosin-I structures organized in a ring at the pseudopod tip [31]. As the fibre is drawn in, cytoskeletal stiffening would allow it to slide along the cell membrane. We hypothesize that in tubular pseudopods, stress filaments would contract asymmetrically (Fig. 3b), with one side contracting and the opposite side expanding. This differential action would produce a rotational force via the Poisson effect. In consequence, fibres enclosed within the pseudopod would become bent, with their curvature determined by the balance between this rotational force and the fibre’s intrinsic rigidity.

Stress filaments consist of actin, myosin-II dimers, and associated crosslinkers (Fig. 3c) [32–34]. Their contractions result from myosin-II–driven sliding of actin filaments, producing compressive stresses whose magnitude depends on the number of participating actomyosin structures. Building on this hypothesis, contractile forces were estimated and transformed into bending forces. See Appendix A for a detailed description. A single stress filament could generate bending forces within the range of sub-nanonewton to nanonewton magnitudes, comparable to pulling forces reported during early phagocytosis [31]. For a complete picture of interacting stress filaments along the pseudopod, we finally hypothesize that their contractile capacity would depend on the number of activated myosin-II dimers, determined by local ATP availability. Since ATP levels are known to decrease along cellular protrusions with distance from the base [35, 36], we assumed a gradient in contractile strength along the pseudopod, resulting in a spatially declining bending force described with a simple hyperbolic profile (see Eq. 4 in Appendix A).

### Formulation of a pseudopod bending model

We developed a mathematical model of the pseudopod’s cytoskeletal nanomachinery based on the hypothetical bending mechanism described above to examine how different boundary conditions affect fibre deformation dynamics and resulting shapes. The mathematical formalism is described in detail in Appendix B. A comparable configuration of force-generating elements exists in robotic bending arms that employ pneumatic actuators [37–39]. Multiple actuators are arranged along a straight structure to enclose a flexible beam, resembling the stress filaments in a pseudopod. Once the structure buckles, the actuators generate a force towards the curvature centre, i.e., perpendicular to the local tangent—a so-called follower force. Beam deflections caused by such actuators are commonly described using the Euler–Bernoulli theory [40]. The resulting solutions define the deflection curve, i.e., the shape of the beam under transverse load. The model generates deflection curves influenced by parameters such as the hyperbolic bending force profile, the pseudopod length that defines the arcing portion of the fibre, and its rigidity. Such curves are compared with observed fibre shapes during and after dynamic bending to evaluate the model’s validity and estimate fibre rigidity.

**Fig. 4:**
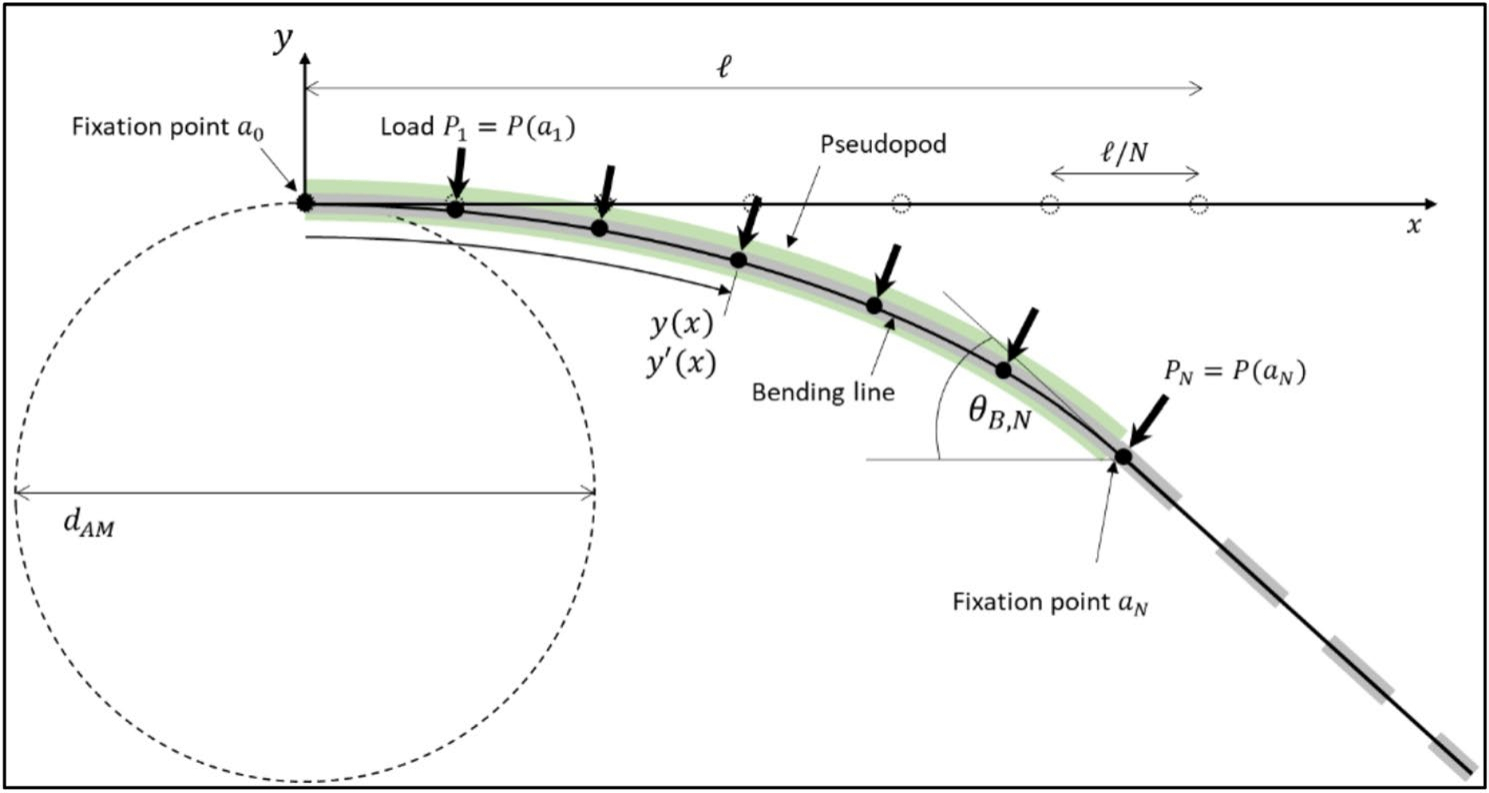
Bending model used to simulate fibre bending by a macrophage. The NR8383 macrophage is represented by a circle of diameter d_AM_ at which the fixation point a_0_ of a pseudopod of length $\ell$ is placed. Bending by individual stress filaments occurs at *N* positions points *a*_*1*_, …, *a*_*N*_ distributed equidistantly along $\ell$. A discrete force distribution P(a_1_, …, a_N_) is applied at the anchor points, with the case of *N* = 6 being shown. The force always applies perpendicular to the local fibre segments. This way, the fibre is bend into an arc, with the slope *y’*(*x*) determining the bending line *y*(*x*). Local angle of deflection $\:{\theta}_{B,N}$(*x*) opens up between x-axis and tangent of the local fibre segment. An iterative approach to solve the bending system was employed that is described in more detail in Appendix B

**Fig. 5:**
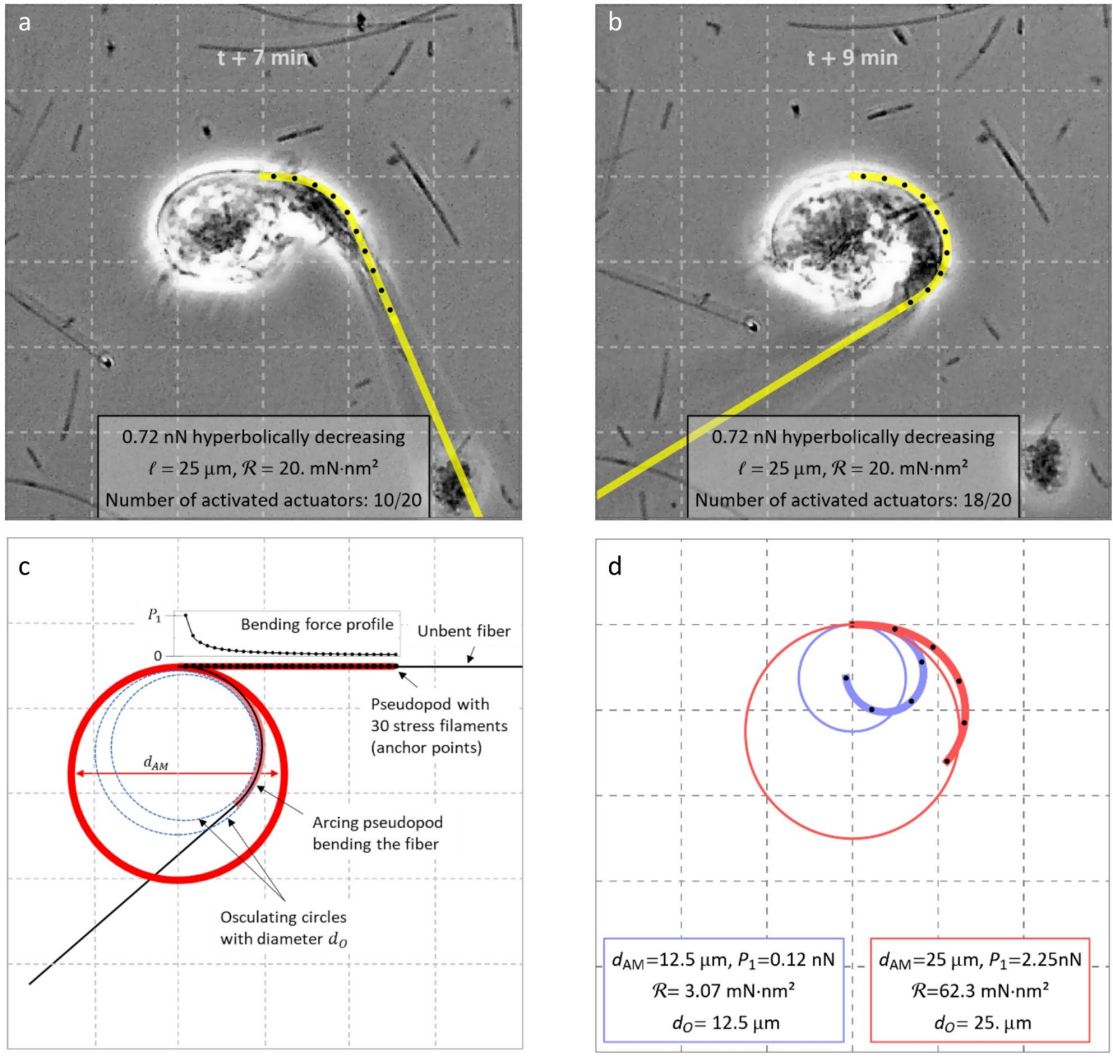
Application of the bending model to simulate observed bending dynamics and predict critical rigidity range. **a**,** b**, Deflection curves calculated with the iterative bending model overlaying the microscopical images, which were rotated so that the fibre near the pseudopod base aligned horizontally. In all calculations, anchor points were distributed over the maximum observed pseudopod length of 25 μm, only every 2nd being shown as black dots. The grid applies to both cell images and plots. Each grid line represents a distance of 10 μm. The force profile was determined using the average values for the stress filament architecture as discussed in Material and Methods: *N*_*myo*_ = 20, *N*_*actin*_ = 20 and *f*_*myo*_ = 4 pN and $\:\nu\:$ = 0.45, which yields *P*(*a*_*1*_) = *P*_*1*_ = 0.72. **a** and **b** show the bending event of a fibre with a rigidity of $\:\mathcal{R}=$20 mN·nm² seven and nine min after initialization of phagocytosis at time *t*, respectively, overlayed by calculated deflection curves in yellow with bending applied respectively to the first 10 and 18 anchor points, respectively. The exterior unbent section of the fibre is represented by the tangent at the last anchor point. **c**, Concept of determination of critical rigidity. The red circle denotes the cell, black lines the fibre in unbent and bent states, and red lines the pseudopod in straight and arced form. Black dots along the pseudopod represent stress filaments serving as anchor points for the applied bending force. A hyperbolic force profile is plotted (see Appendix A), each value corresponding to the anchor point below. Osculating circles (blue) fitted to the bending line near anchor points are shown for front and rear fibre sections. The average osculating circle fits within the cell, indicating successful bending and a rigidity below the assumed critical threshold. **d**, Boundary cases enabling definition of a critical rigidity range. For the blue case, the lower bound of the bending force and the reported cell size for the inactive macrophage was used, the upper bound of the bending force and observed maximum distorted cell size for the red case. A critical rigidity range of ca. 3–62 mN·nm² can be predicted

The fibre is represented by a cantilever beam encompassed by the tubular pseudopod that deforms, thus exerting shear forces causing the fibre to bend (Fig. 4). Two boundary conditions determine the model: fixed at one end, i.e., the pseudopod base, and free to rotate at the tip. We observed that the fibre is bent with approximately constant curvature (Fig. 1b, t + 8 min), indicating a nearly uniform bending moment along its length. For a Euler-Bernoulli beam of constant diameter, such profile cannot result from a uniform load, but requires a non-uniform one, for example the application of spatially discrete but decreasing forces as described in Eq. 4 (Appendix A). This sequential application of forces at discrete anchor points served as an approximation of the pseudopod’s bending dynamics. Deflection curves overlaying the microscopical images of bent fibre segments were determined when forces were applied to the first 10 and 18 anchor points (Fig. 5a, b), representing the state during and at the end of bending. The force profile was modelled using the average values for the stress filament architecture as discussed in Appendix A, resulting in an application of 0.72 nN at the first anchor point. A rigidity of 20 mN∙nm² yielded the best match to the observed arcs of the fibres in the bending sequence. The equivalent fibre diameter for silver nanowires would be ca. 47 ± 3 nm, a value near the modal width of the measured width distribution for the critical fraction of aged Ag-Rods-3170 nanowires (Supporting Information Figure S1a). A force profile as described earlier, determined by myosin-II activity and linearly decreasing with pseudopod length, would not result in a unform curvature, but in curvature increasing with length, eventually leading to a spiralling fibre. Our simulation showed that fibres bent by a pseudopod with quadratically or cubically decreasing myosin-II activity evolved an almost uniform curvature. However, information about actual myosin-II activity over the pseudopod length is currently unknown. Thus, the simple linear case is used for further discussion.

### Estimation of a critical rigidity range

A critical condition distinguishing successful from unsuccessful fibre uptake is that a long fibre bends into an arc fitting within the macrophage (Fig. 5c). Osculating circles were applied at the anchor points apart from the first and last, with the mean of their diameters *d*_*O*_ in comparison with the cell diameter *d*_*AM*_ serving as a criterion. If smaller, the bent fibre would fit into the cell. To estimate limits for a rigidity range allowing successful bending, we applied our bending model for borderline values for both maximum bending force *P*_*max*_ and cell diameter. From a qualitative perspective, these scenarios correspond to boundary cases: a “weaker” macrophage generating a tighter fibre curvature (worst case), and a “stronger” macrophage inducing a broader arc (best case). Bounds of the bending force applied to the first anchor point were determined using literature values for the force exerted by each active myosin-II dimer, the number of myosin-II dimers *N*_*myo*_ per actin filament and the number of actin filaments in each stress filament *N*_*actin*_ (Eq. 3 in Appendix A) [35, 36], resulting in *P*_*1*_ = *P*_*max*_ = 0.12–2.25 nN. Note that the reported bounds for *N*_*myo*_ also determined the number of anchor points. Hence for the worst case and best case, *N* = 10 and *N* = 30 was used, respectively. The upper bound for the cell size was estimated to correlate with the maximum cell size observed after the fibre uptake, during which the cell expanded to ca. 25 μm due to the relaxation of the spiral-spring-like fibre. For the lower bound, a typical cell diameter of 12.5 μm was used, often observed for rounded-up inactive macrophages. Resulting bending curves for the two boundary cases were determined (Fig. 5d) where a smaller rigidity of 3 mN∙nm² would allow the smaller and “weaker” cell to take up the fibre and a fibre with a larger rigidity of 62 mN∙nm² was still flexible enough for the larger and “stronger” macrophage. Taking this into account, a critical rigidity can be assumed to be within the range of $\:{\mathcal{R}}_{cr}=$3–62 mN∙nm². This range includes most of the measured rigidity values for the critical fraction of aged silver nanowires of 23 $\pm _{{14}}^{{26}}$ mN∙nm². In addition, this rigidity range is similar to the bounds of the critical rigidity value of 7 $\:{\:}_{-6}^{+31}$ mN∙nm² for MWCNTs, deduced from Rittinghausen et al. [25]. This value range applies to a critical diameter range of 30 ± 2 nm to 63 ± 4 nm for silver nanowires.

A single fibre-bending event was observed during the course of the experiments, which limits the validity of the present model. Subsequent experimental refinements may enhance the probability of identifying and systematically analyzing additional fibre–cell interactions. The rigidity hypothesis is applicable to a range of fibre materials; therefore, alternative fibres should be selected that exhibit comparable rigidity distributions, but with a higher proportion of long fibres (> 50 μm). Polymer-based fibres constitute a particularly suitable class of materials, as their morphological properties can be systematically and precisely tailored [41]. Such fibres typically possess bending moduli in the order of 1 GPa, corresponding to a critical diameter range of approximately 88–189 nm for the estimated critical rigidity range. These fibres can be fabricated by electrospinning [42] and subsequently processed to obtain homogenized fibre length distributions [43]. Moreover, modern phase-contrast microscopy enables larger fields of view at comparable magnifications, thereby increasing the number of cells that can be observed simultaneously without compromising the spatial resolution required to resolve fibre phagocytosis dynamics.

A precise understanding of the stress filament network within the macrophage pseudopod and a more accurate estimation of the compressive forces generated by its actomyosin structures would allow a sharper determination of the critical rigidity range. This estimation could be refined with detailed information on the architecture of the cytoskeletal bending apparatus, which in our model was approximated from literature describing general cytoskeletal nanomachinery. Advanced microscopical techniques such as cryo–transmission electron tomography (Cryo-TEM) [44] enable visualization of biomolecular structures without disturbing their native organization. Such analyses would improve the bending model by providing more realistic force profiles and, consequently, more accurate predictions of the critical rigidity range.

Our use of a rat alveolar macrophages restricts the ability to make direct extrapolations to human physiology. On average, human alveolar macrophages are larger than rat alveolar macrophages, with mean diameters of approximately 21.2 ± 0.3 μm and 13.1 ± 2.5 μm, respectively [28, 45]. These differences in cell size, together with potential differences in cytoskeletal organisation and force-generating machinery, may influence the fibre length at which frustrated phagocytosis occurs, as well as the forces available for fibre bending. This would affect the critical rigidity range. While adherent NR8383 macrophages exhibit pronounced spreading and dynamic extension during fibre interaction, reaching effective interaction diameters of over 50 μm, which may mitigate some of the differences associated with nominal cell size, future studies on human alveolar macrophages (primary or e.g. stem cell-derived equivalents) are required to fully transfer our results to the human lung.

At least until then, a conservative approach by assuming ‘weak’ macrophages allows the estimation of a critical rigidity value of$\:\:{\mathcal{R}}_{cr}=$ 3 mN∙nm² below which a fibre can safely be assumed flexible for the NR8383 cell line.

## Conclusions

Our observations of fibre bending by macrophages support the hypothesis that retention and persistence of a fibre in the lung due to incomplete phagocytosis not only depends on its length but also on its rigidity. Based on the findings and referenced data presented, we propose a NR8383 cell-line-specific rigidity threshold of about 3 mN·nm². This result cannot be applied directly for human physiology and additional experiments are required to validate the rigidity hypothesis for human alveolar macrophages as well as establish a respective critical rigidity threshold, best using human primary alveolar macrophages.

This threshold abstracts from the underlying material-specific critical diameters. Fibre rigidity should be considered a defining criterion of toxicologically critical fibres, complementing respirability, biodurability, and length. We hope that an extended fibre-pathogenicity paradigm will lead to refined regulatory frameworks and guide the design of nanoscale fibre materials of low inhalation risk.

It is important to note that flexural rigidity itself cannot be measured directly. It is imperative to recognise the necessity of converting routinely measured fibre width distributions into corresponding rigidity distributions. This process can be facilitated by utilising Eq. 1, under the presumption of a known bending modulus. The implementation of this method is a crucial aspect in the rigorous examination of the rigidity hypothesis. A key challenge is the paucity of quantitative bending modulus data, which may differ substantially from the more commonly reported Young’s modulus, which reflects tensile or compressive stiffness rather than flexural behaviour. In order to facilitate robust, rigidity-based comparisons across a range of fibre materials and to underpin future regulatory frameworks that consider fibre rigidity as an additional hazard descriptor, it is recommended that progress is made in the field of bending modulus metrology for nano- and microscale fibres. This would allow for the systematic characterisation of fibre materials using techniques such as three-point bending [46] or resonance frequency methods [22]. While the width distributions must be determined for individual fibre batches, the bending modulus values can, in most cases, be transferred across materials with an equivalent substance base. This is essential for translating the rigidity hypothesis into practical and regulatory-relevant fibre hazard assessments.

## Supplementary Information

Below is the link to the electronic supplementary material.

Supplementary Material 1

## Figures and Tables

**Fig. 6:**
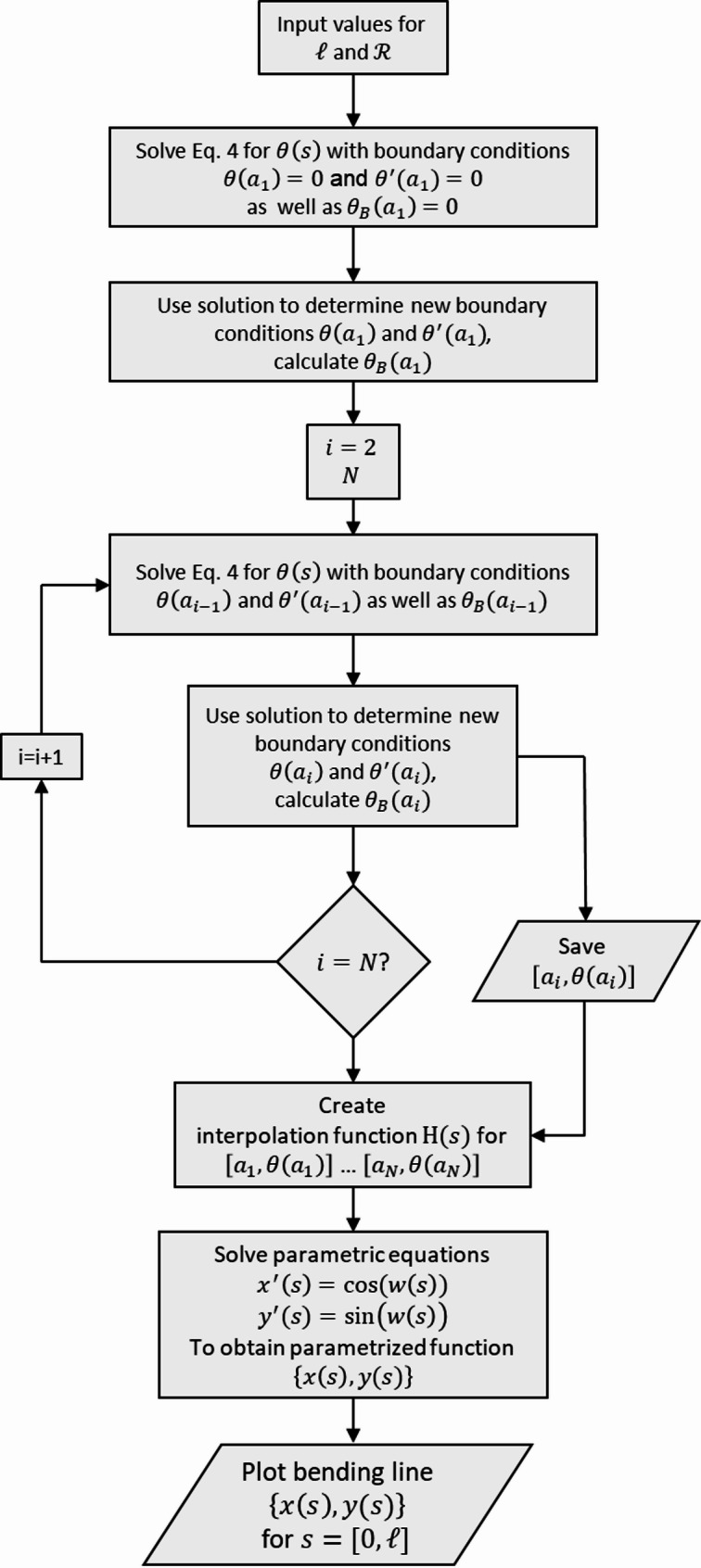
Flow scheme of the iterative determination of the deflection curve representing the arcing fibre section subject to a bending pseudopod

## Data Availability

No datasets were generated or analysed during the current study.

## Appendix A

### Force estimation for fibre bending

To estimate the forces exerted on fibres during phagocytosis, we applied a simplified biophysical model of pseudopod contraction. Bending of a fibre enclosed in the tubular pseudopod is assumed to result from asymmetric contraction of stress filaments, producing a rotational force *P* via the Poisson effect. The Poisson effect describes lateral strain induced by a uniaxial compressive stress. Accordingly, the rotational force is given by:

2 $$ \:P = \nu \: \cdot F_{{{\mathrm{comp}}}} $$

where *F*_*comp*_ is the compressive force generated by the stress filaments and $\:\nu\:$ is the Poisson ratio. For cytoskeletal networks, values of $\:\nu\:$=0.4–0.5 have been reported [47, 48].

Stress filaments consist of actin filaments crosslinked by myosin-II dimers and accessory proteins [32, 33]. The compressive force they generate depends on the number of actin filaments within a stress filament (*N*_*actin*_), the number of myosin-II dimers interacting with each actin pair (*N*_*myo*_), and the average contractile force generated by one myosin-II dimer (*f*_*myo*_=3–5 pN) [34]. The compressive force can thus be estimated as:

3 $$\:{F}_{\mathrm{c}\mathrm{o}\mathrm{m}\mathrm{p}}=\:{N}_{\mathrm{a}\mathrm{c}\mathrm{t}\mathrm{i}\mathrm{n}}\:{N}_{\mathrm{m}\mathrm{y}\mathrm{o}}\:{f}_{\mathrm{m}\mathrm{y}\mathrm{o}}.\:$$

Reported values for stress filaments range between 10 and 30 actin filaments [35], with each actin filament hosting 10–30 myosin-II dimers [36]. These parameters yield bending forces in the range of 0.12–2.25 nN per stress filament, which is comparable to the maximal pulling forces (~ 3 nN) reported for phagocytosis [31].

Since ATP supply would decrease with distance from the pseudopod base [49, 50], we assumed that the number of activated myosin-II dimers declines linearly along the pseudopod length. With equidistant stress filaments positioned at locations *a*_*1*_, …, *a*_*Nmyo*_ and the maximum number of activated dimers *N*_*myo*_​ at the base, the resulting force profile is described by:

4 $$ \begin{aligned} \:P\left( {a_{n} } \right) = \nu \: \cdot \:F_{{comp,\:\:n}} \\\quad = N_{{actin}} \:\frac{{N_{{{\mathrm{myo}}}} }}{n}\:\:f_{{{\mathrm{myo}}}} \:\:\:\:\:\:\:\:\:\: \\\quad {\mathrm{with}}\:n = 1,\: \ldots \:,\:N_{{{\mathrm{myo}}}} \: \\ \end{aligned} $$$$ $$

This formulation yields a hyperbolic decline in contractile force along the pseudopod length.

## Appendix B

### Implementation of the iterative bending model

Anchor points *a*_*n*_ are defined along the fibre section subject to the pseudopod of length $\ell$:

5 $$ \:a_{n} = n \cdot \:\left( {\ell /N} \right) $$

where *N* is the total number of anchor points, representing the number of locations over the pseudopod at which contraction and expansion of the cytoskeletal network happens.

The Euler Bernoulli beam equation can be expressed in terms of the slope *y’(x)* of the beam as:

6 $$ \:{\mathcal{R}}\:y^{{\prime \:\prime \:\prime \:}} \left( x \right) = - q\left( x \right) \cdot \:{\mathrm{cos}}\left( {\theta \:_{B} \left( x \right) - {\mathrm{y}}\prime \:\left( x \right)} \right) $$

where *q(x)* is the local force applied and $\:{\theta}_{B}$(x) is the local bending angle. Before bending, $\:{\theta}_{B}$(x) = 0 for the case of an unbent fibre. This is a second-order differential equation that can be solved by setting boundary conditions for the fixed end.

Boundary conditions are given at the previous anchor point *a*_*n−1*_ at which both the slope *y’* and the local curvature *y’’* is fixed as a result from the previous bending.

7 $$\:y{\prime\:}\left({a}_{n}\right)=\mathrm{y}{\prime\:}\left({a}_{n-1}\right)$$

8 $$\:{y}^{{\prime\:}{\prime\:}}\left({a}_{n}\right)=\frac{dy{\prime\:}}{dx}\left({a}_{n}\right)=\frac{dy{\prime\:}}{dx}\left({a}_{n-1}\right)$$

With the help of Mathematica, iterative bending was implemented, described here in a flow chart (Fig. 6). At the beginning, values for $\ell$ and $\:\mathcal{R}$ are defined. Equation 4 is solved by using the initial boundary conditions *y’*(*a*_*1*_) = 0 and *y’’*(*a*_*1*_) = 0, which represents the unbent fibres with zero rotation and zero curvature. The bending force is applied onto the anchor point $\:{a}_{2}$ with initial angle $\:{\theta\:}_{B}$ (*a*_*1*_) = 0. The solution gives the slope *y’*(*a*_*2*_) as well curvature *y’’*(*a*_*2*_), used as boundary conditions for the next iteration. A new bending angle is given by the angle of slope at location $\:{a}_{2}$: $\:{\theta\:}_{B}\left({a}_{2}\right)={\mathrm{tan}}^{-1}\left({y}^{{\prime\:}{\prime\:}}\left({a}_{2}\right)\right)$. This process is repeated until the last anchor point $\:{a}_{N}$ is reached. This way, data points of [*a*_*1*_, *y’*(*a*_*1*_)] to [*a*_*N*_, *y’*(*a*_*N*_)] are generated, essentially a list of slopes at the anchor points that can be used to find a cubic Hermite interpolation *H*(*s*) function fitting the data points.

9 $$\:H\left(s\right)=a\:s+b\:{s}^{2}+c\:{s}^{2}$$

The interpolating function is then used to parametrize the slope of the deflection curve:

10 $$\:xx{\prime\:}\left(s\right)=\mathrm{cos}\left(H\left(s\right)\right)$$

11 $$\:yy{\prime\:}\left(s\right)=\mathrm{sin}\left(H\left(s\right)\right)$$

The parametrized deflection curve in cartesian coordinates {*xx*(*s*), *yy*(*s*)} is then determined by finding the integral of *xx’*(*s*) and *yy’*(*s*) within the bounds *a*_*1*_ and *a*_*N*_.

One can implement a parent process iteratively increasing *N* which would represent the consecutive “switch-on” of the bending anchor points.
