# Supplementary material for: Macrophages bend long fibres with flexural rigidity lower than 3 mN·nm2 to avoid frustrated phagocytosis
Source: Part Fibre Toxicol. 2026 Mar 18;23:15. doi: 10.1186/s12989-026-00666-9 (PMC12998087; doi:10.1186/s12989-026-00666-9)
Supplement: Supplementary file 1 — Supplementary Material 1 [file 12989_2026_666_MOESM1_ESM.docx]

**Supporting Information**

- **Supplementary video 1**: Time-lapse video of a NR8383 macrophage taking up, bending and internalizing a long fibre of the aged Ag-Rods-3170 nanowire variant.
- **Supplementary video 2**: Time-lapse video of NR8383 macrophages failing to take up rigid long fibres of the fresh Ag-Rods-3170 variant.
- **Supplementary video 3**: Time-lapse video of NR8383 macrophage fully taking up fibres of the Ag-NWs-40 variant.

Please use this link to access the folder containing the videos:

<https://drive.google.com/drive/folders/1wETCUckLaUc3gP_buGf95EIKj1ZhQa35?usp=sharing>

- Figure S1:

| a |  |  |
| --- | --- | --- |
| 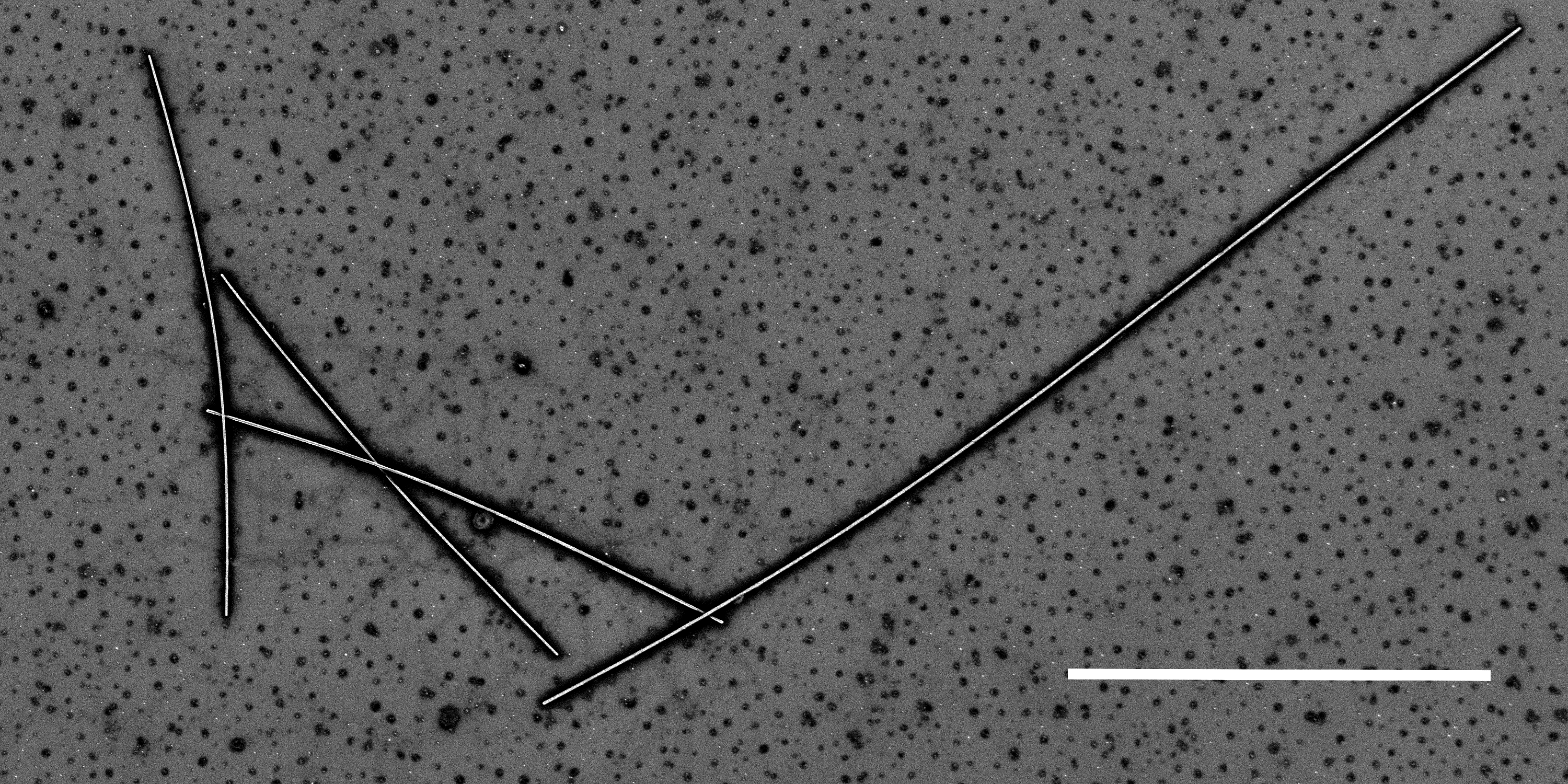Fresh Ag-Rods-3170 | 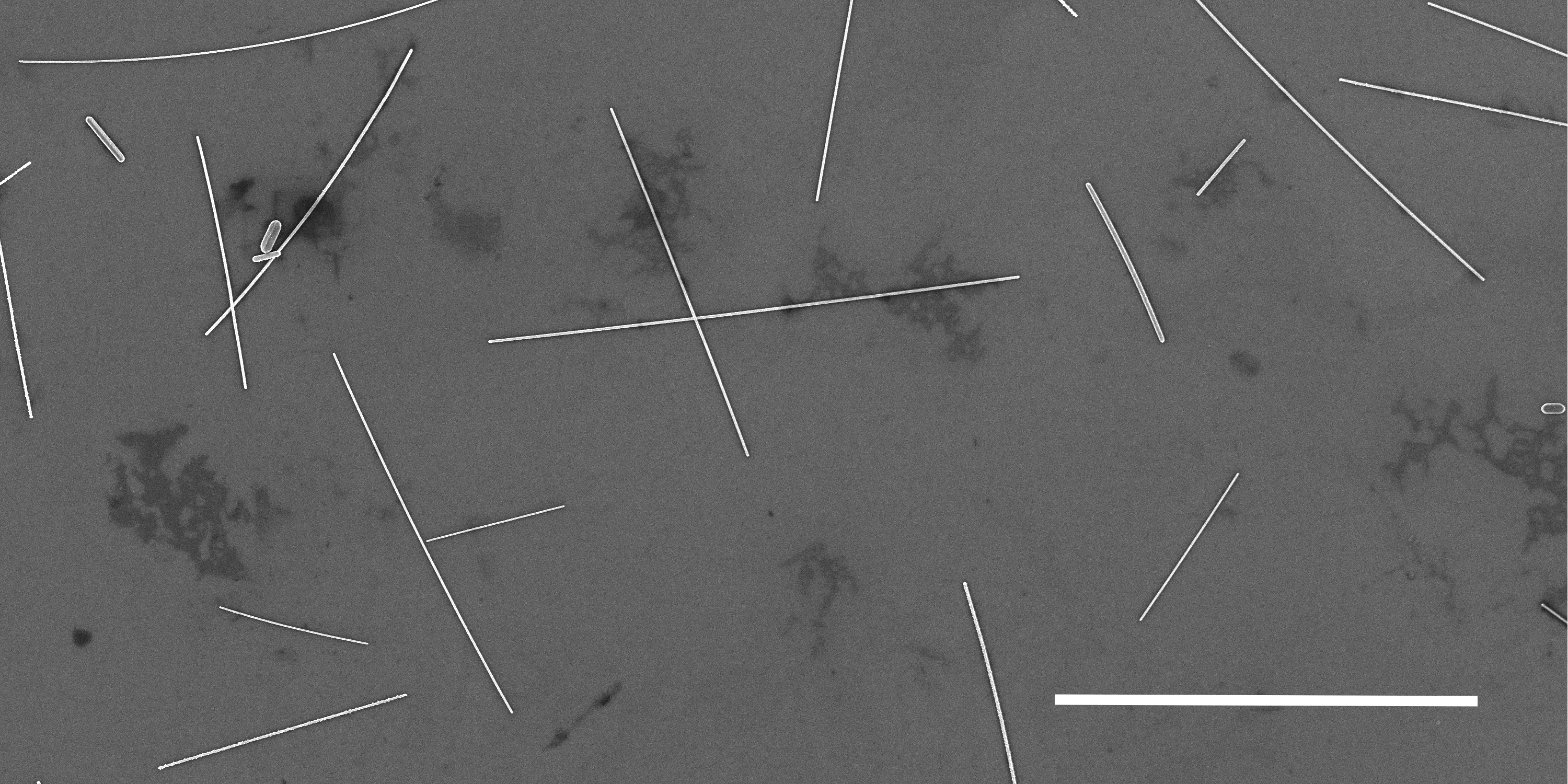Aged Ag-Rods-3170 | 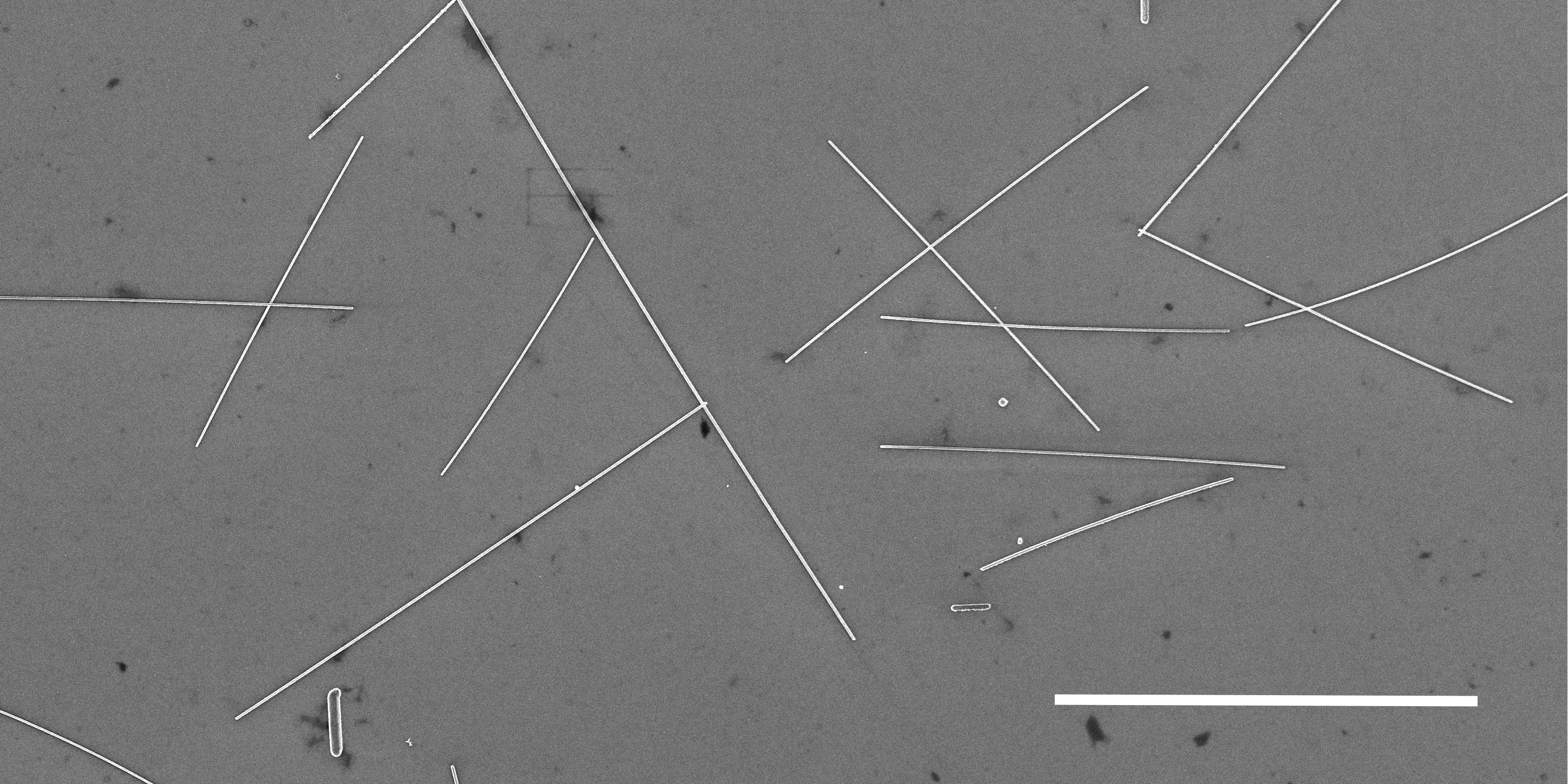Ag-NWs-40 |
| b |  |  |
| 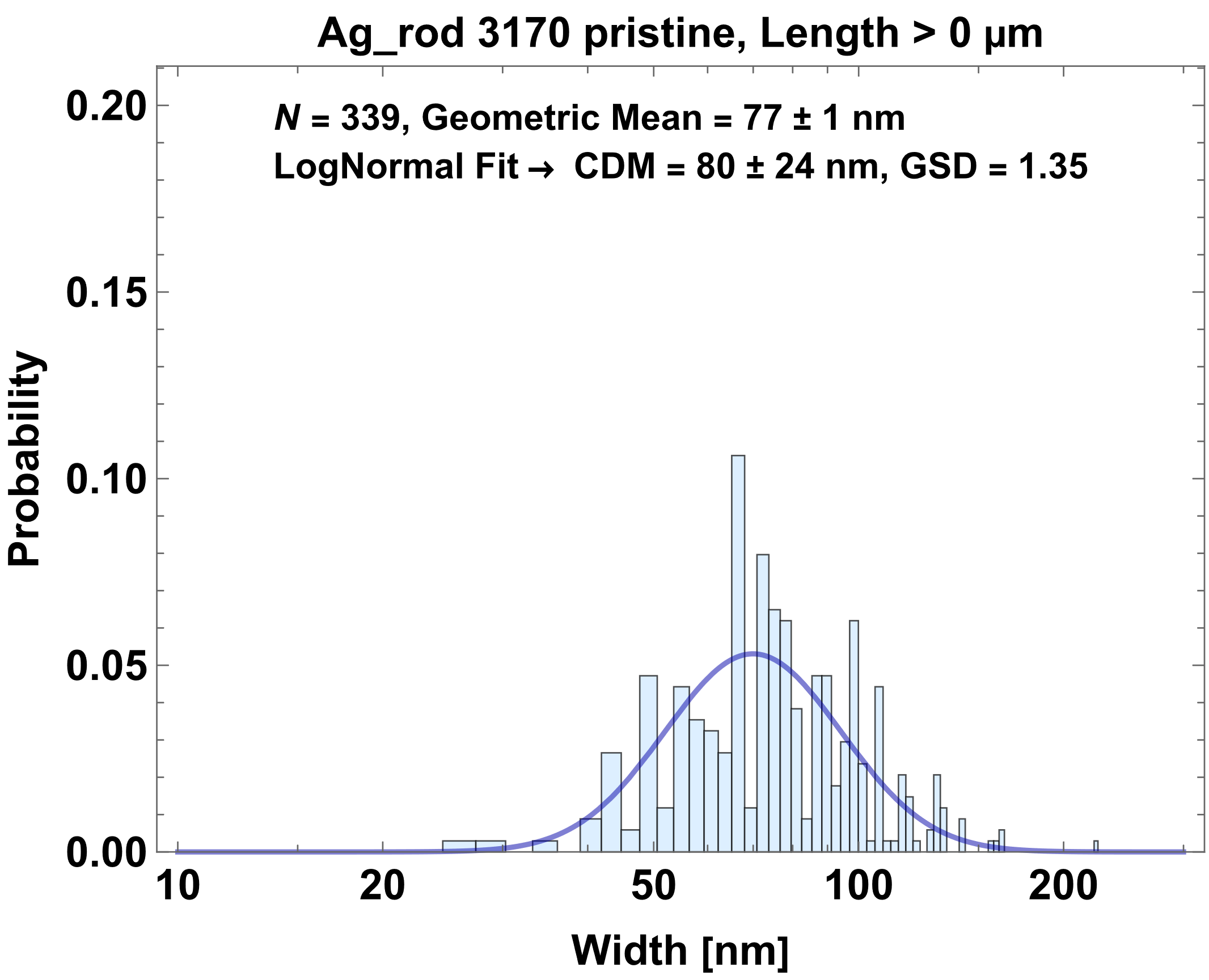 | 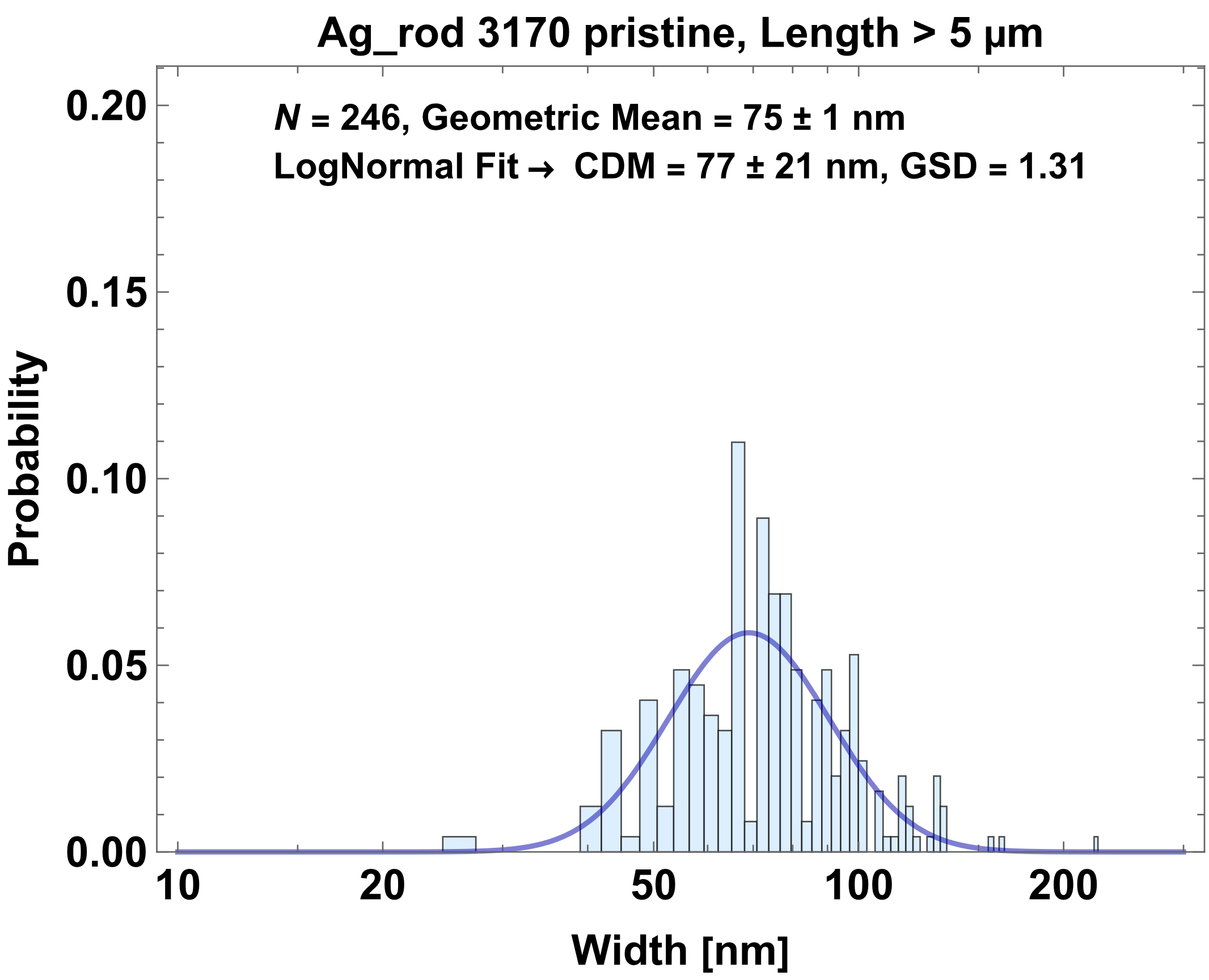 | 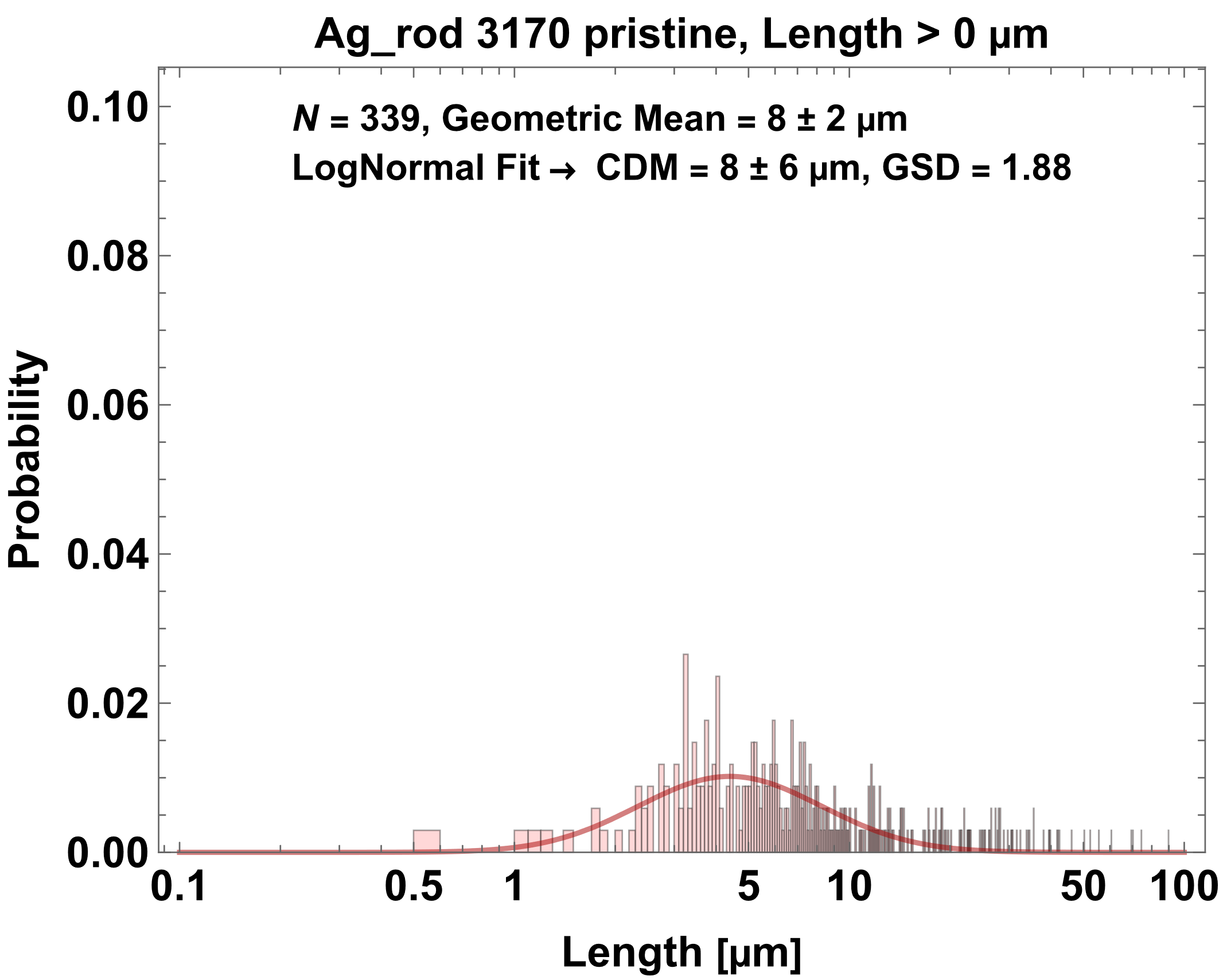 |
| c |  |  |
| 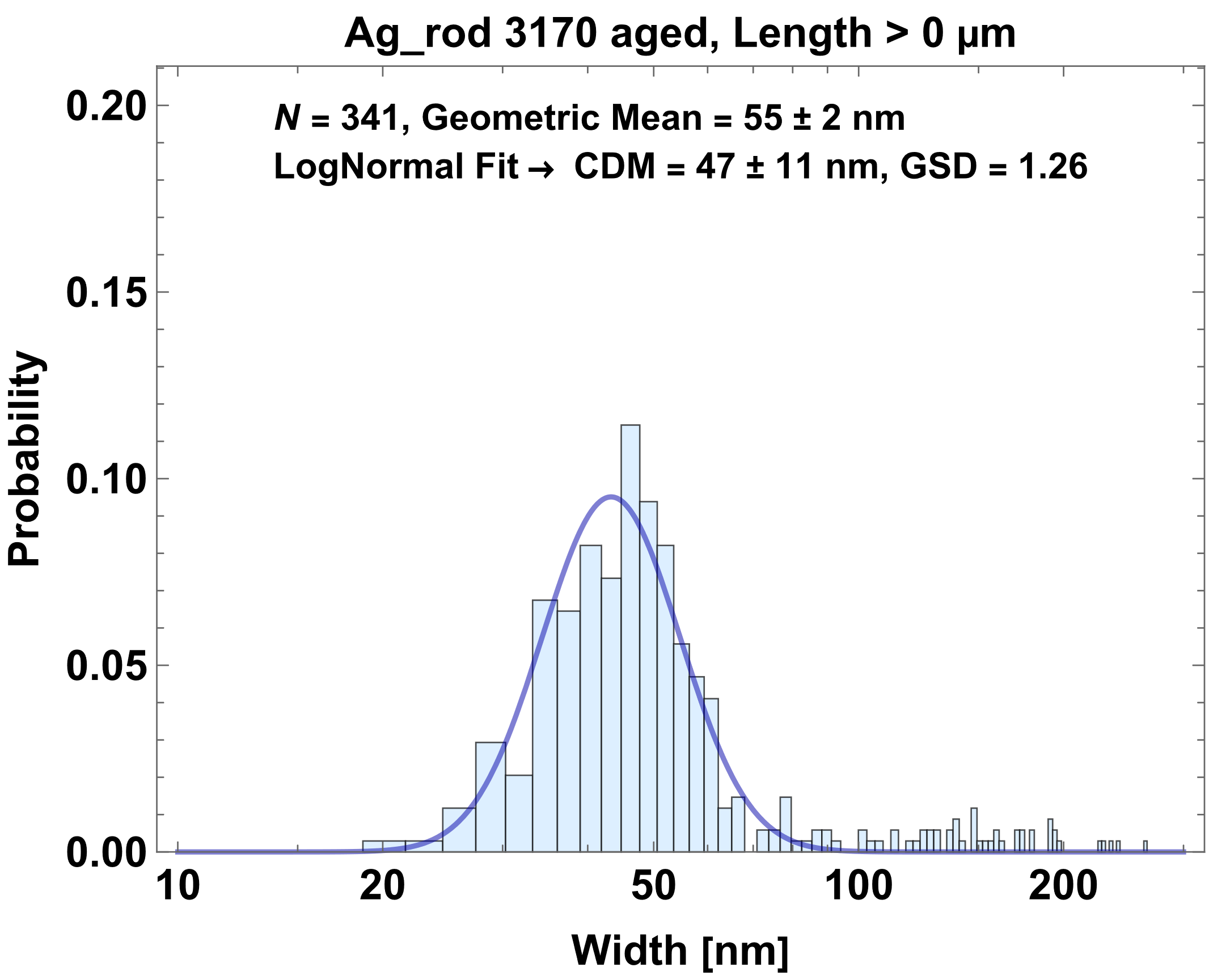 | 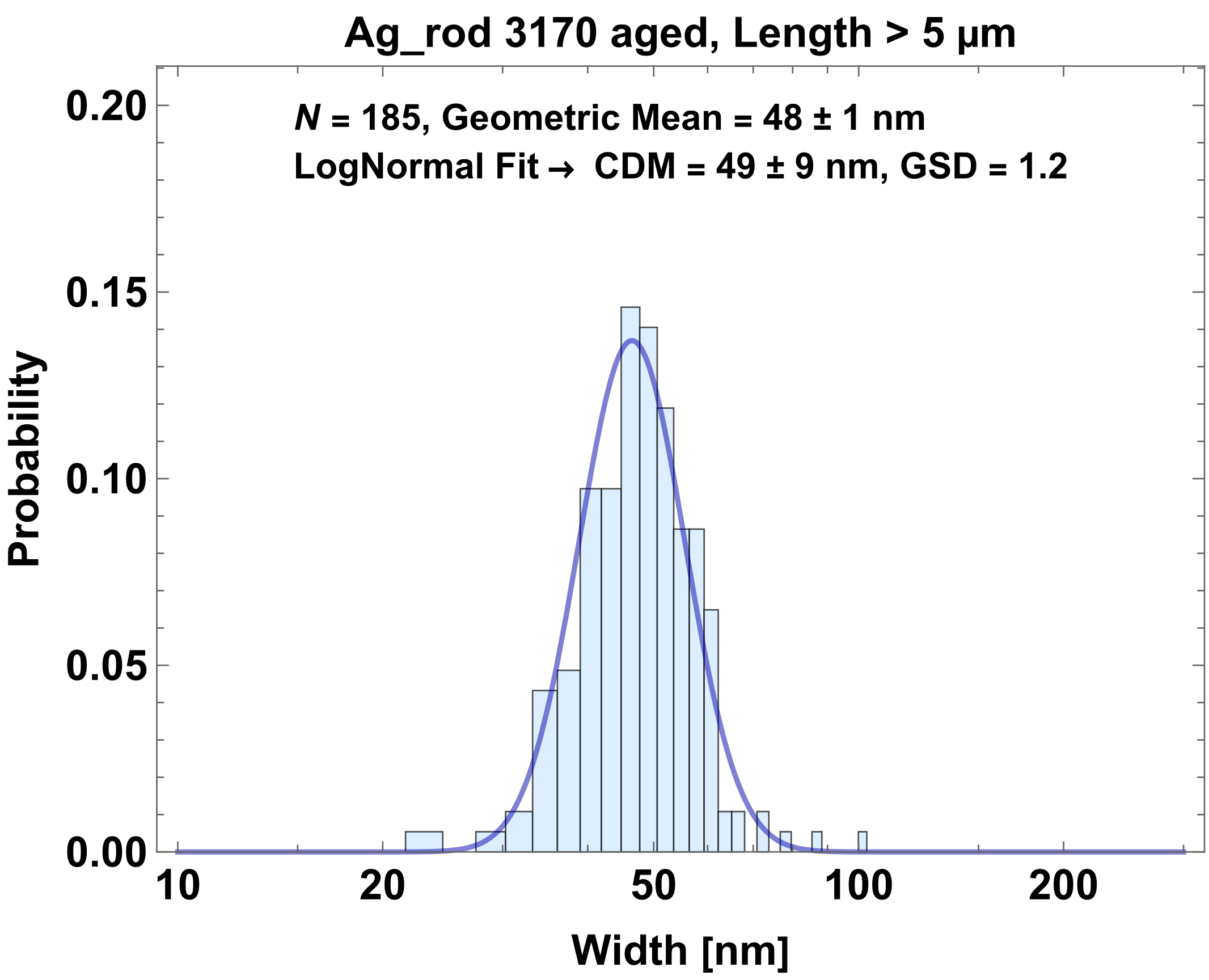 | 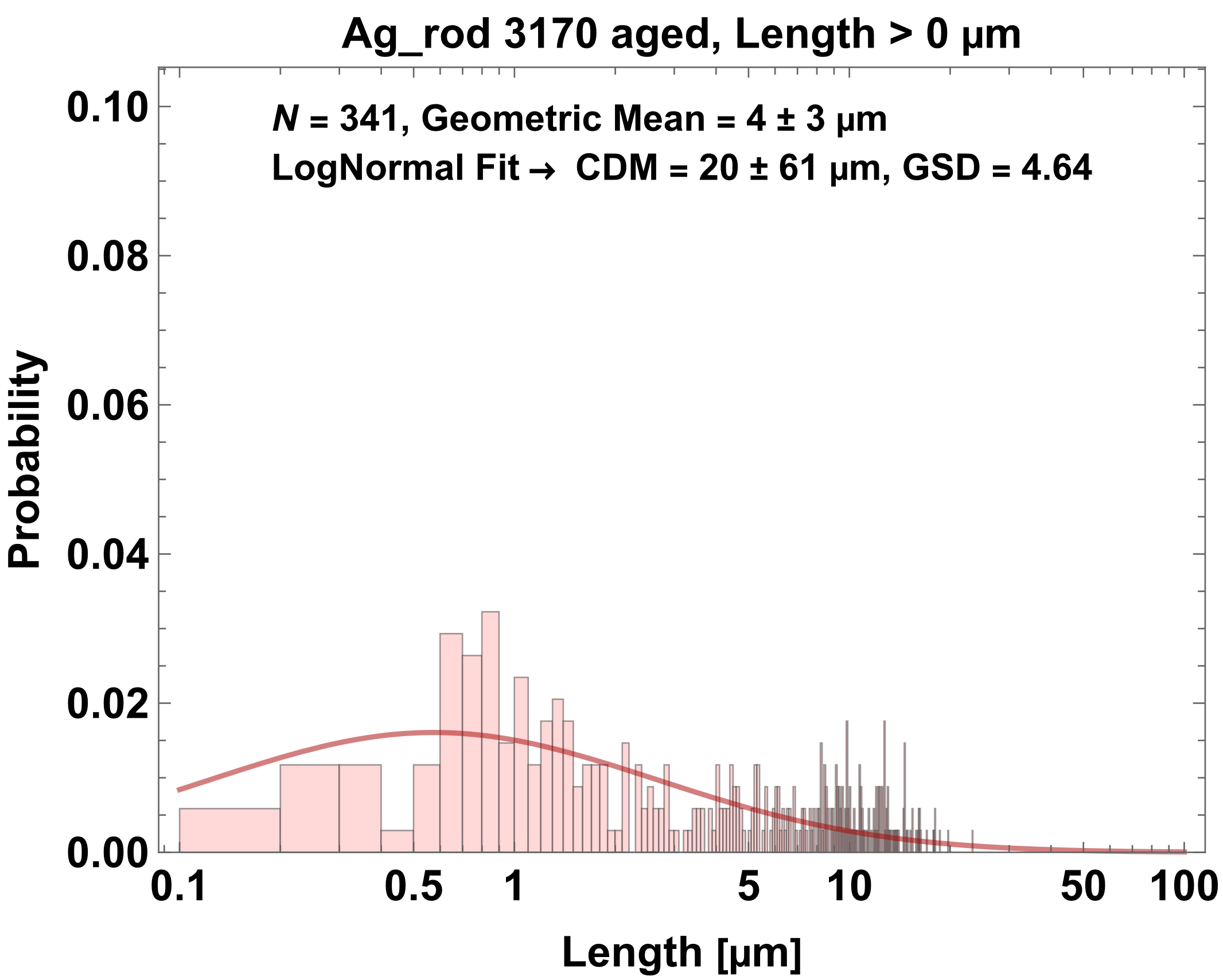 |
| d |  |  |
| 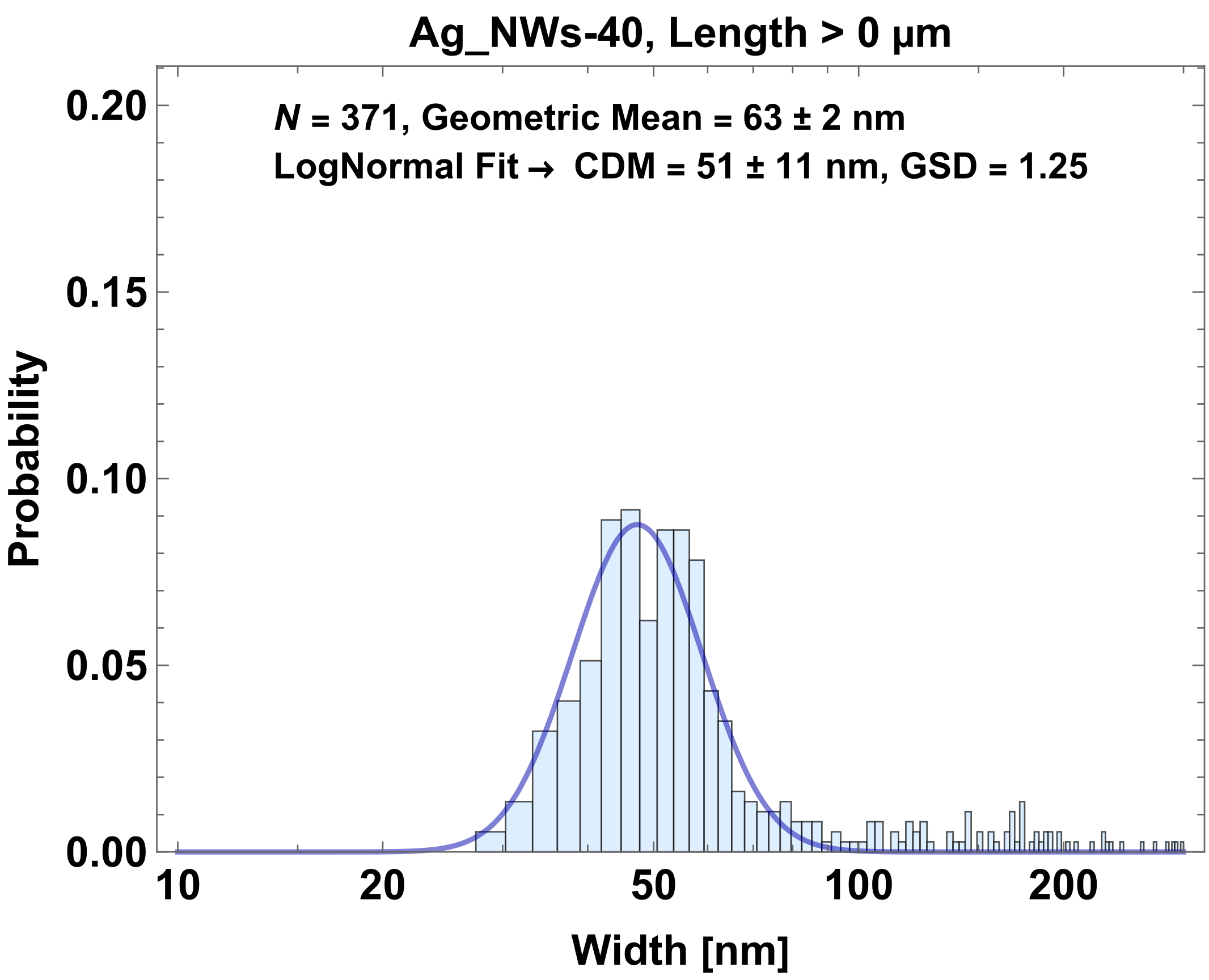 | 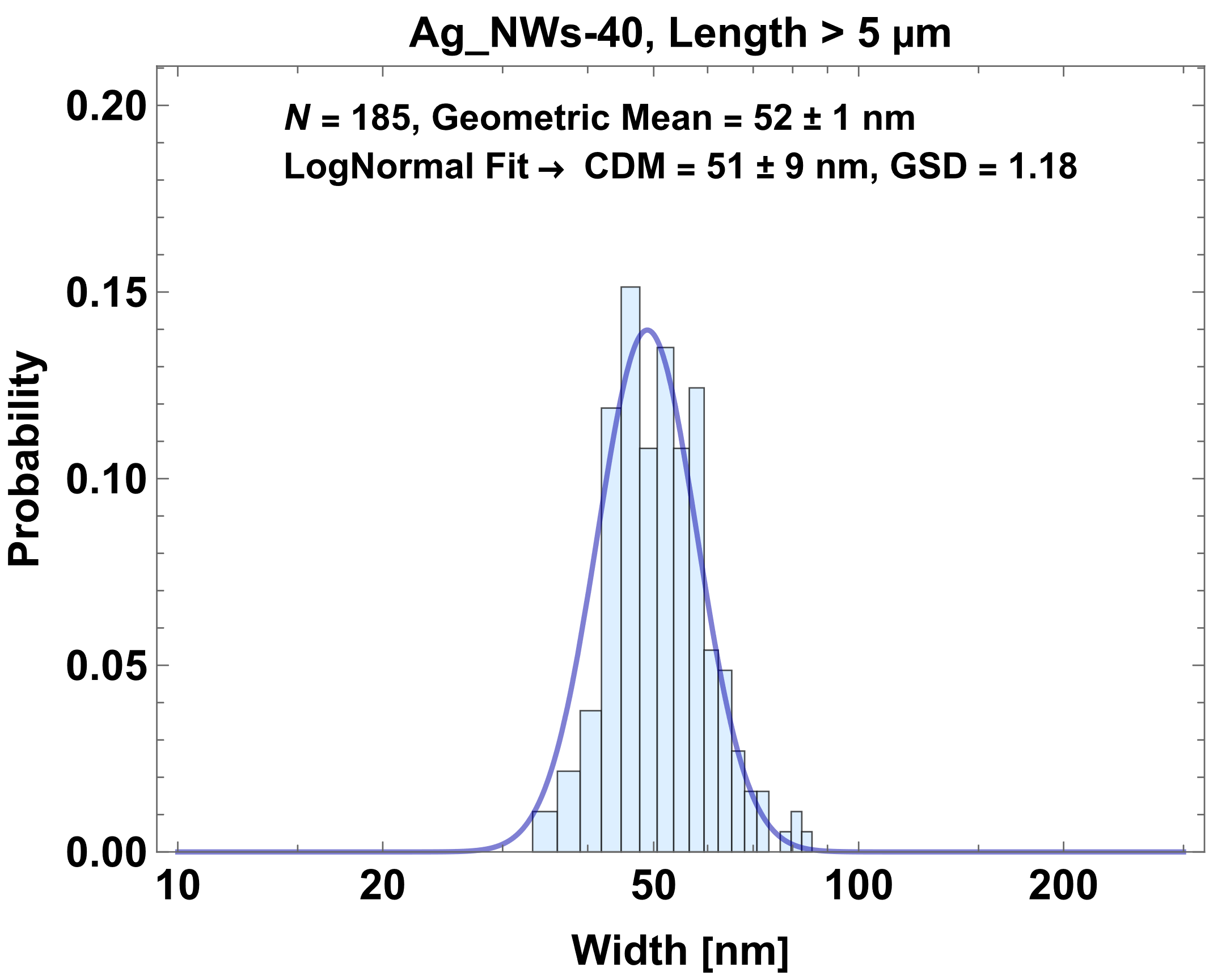 | 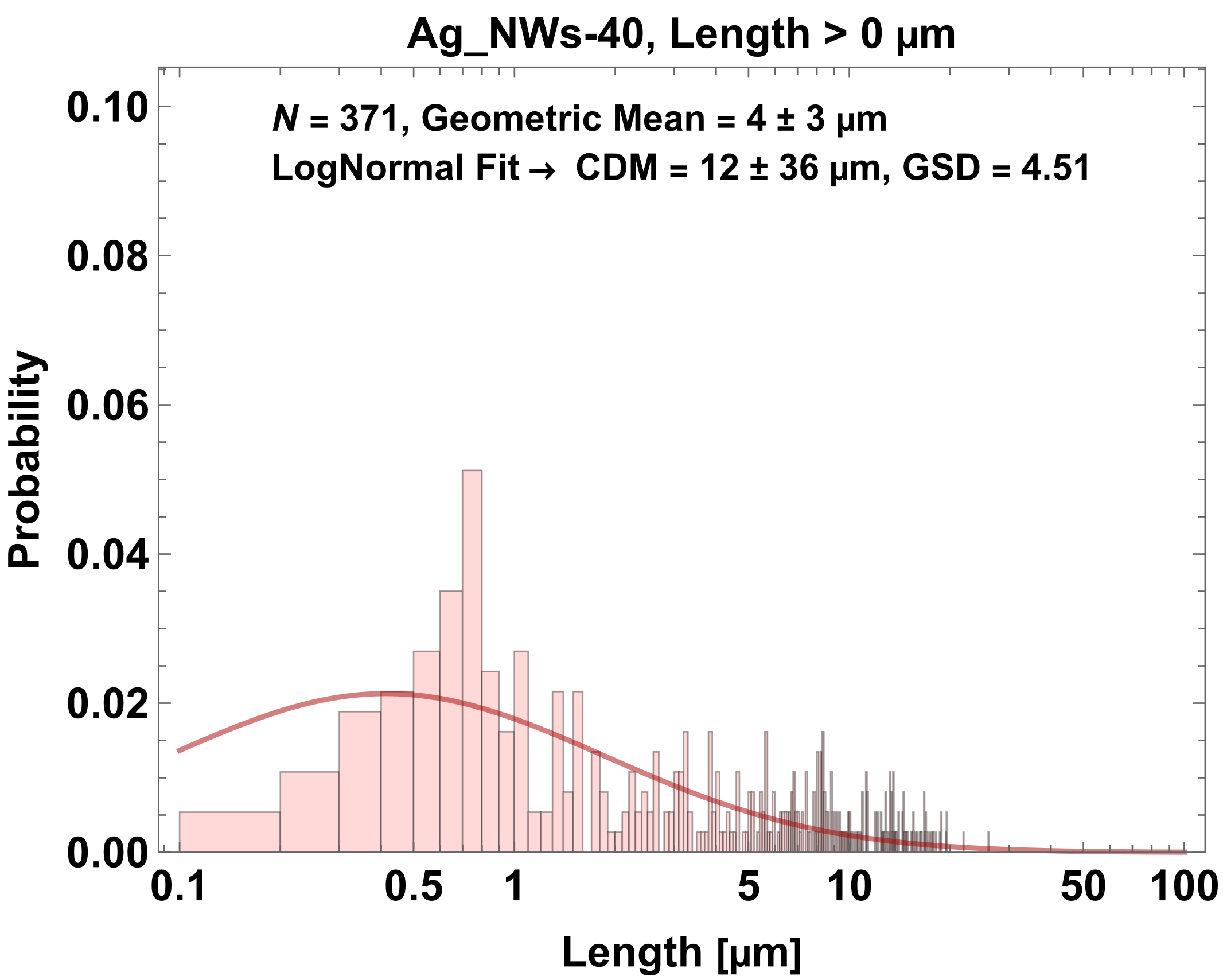 |

**Figure S1.** Morphological characterization of used silver nanowires**. a**, Scanning electron micrographs of fibres deposited on a Si-wafer after dispersion the initial suspensions (see Methods). Scalebars represent 10 µm. **b,c,d**, Width distributions of a sample of all fibres and a subsample of critical fibres (L > 5 µm) as well as length distribution of a sample of all fibres. Widths and lengths were measures pair-wise followed the rules of OECD TG 125 [27]. Histograms were generated counting in 100 bins (width) and 1000 bins (length) with uniform bin sizes. Curves represent probability density functions of the lognormal distribution used to fit the histogram datasets.
